# Synergistic prostaglandin E synthesis by myeloid and endothelial cells promotes fetal hematopoietic stem cell expansion in vertebrates

**DOI:** 10.15252/embj.2021108536

**Published:** 2022-08-04

**Authors:** Pietro Cacialli, Marie‐Pierre Mailhe, Ingrid Wagner, Doron Merkler, Rachel Golub, Julien Y Bertrand

**Affiliations:** ^1^ Department of Pathology and Immunology, Faculty of Medicine University of Geneva Geneva 4 Switzerland; ^2^ Unité Lymphocytes et Immunité Pasteur Institute Paris Cedex 15 France; ^3^ Division of Clinical Pathology, Department of Diagnostic University Hospitals of Geneva Geneva Switzerland; ^4^ Université de Paris Paris France

**Keywords:** hematopoietic niche, HSCs, PGE2, slco2b1, Development, Haematology, Stem Cells & Regenerative Medicine

## Abstract

During development, hematopoietic stem cells (HSCs) are produced from the hemogenic endothelium and will expand in a transient hematopoietic niche. Prostaglandin E_2_ (PGE2) is essential during vertebrate development and HSC specification, but its precise source in the embryo remains elusive. Here, we show that in the zebrafish embryo, PGE2 synthesis genes are expressed by distinct stromal cell populations, myeloid (neutrophils, macrophages), and endothelial cells of the caudal hematopoietic tissue. Ablation of myeloid cells, which produce the PGE2 precursor prostaglandin H_2_ (PGH2), results in loss of HSCs in the caudal hematopoietic tissue, which could be rescued by exogeneous PGE2 or PGH2 supplementation. Endothelial cells contribute by expressing the PGH2 import transporter *slco2b1* and *ptges3*, the enzyme converting PGH2 into PGE2. Of note, differential niche cell expression of PGE2 biosynthesis enzymes is also observed in the mouse fetal liver. Taken altogether, our data suggest that the triad composed of neutrophils, macrophages, and endothelial cells sequentially and synergistically contributes to blood stem cell expansion during vertebrate development.

## Graphical abstract

Stromal cell collaboration in the embryonic hematopoietic niche preserves production of the stem cell specification factor prostaglandin E_2_.

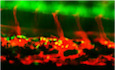

## Introduction

Hematopoietic stem cells (HSCs) are a rare population that can regenerate all blood lineages during fetal and adult life. The original pool of HSCs is established through many developmental processes that involve several specific microenvironments. These hematopoietic niches consist of different cell types, adhesion molecules, and secreted/membrane‐bound signaling factors that can directly affect HSCs or their progeny. A detailed understanding of the complex interactions between HSCs and their niche is therefore critical to improve HSC transplantation‐based therapies (Diez *et al*, 2017; Zonari *et al*, 2017; Gentner *et al*, 2021; Ricci & Cacialli, 2021). While *in vitro* culture systems have allowed a wide comprehension of key signaling involved in HSC differentiation, the mechanisms controlling HSC expansion in steady‐state, which mainly occurs during embryogenesis are still not fully appreciated (Lessard *et al*, 2004). The use of vertebrate model organisms such as mouse and zebrafish has contributed to clarify the mechanisms of hematopoietic development. HSCs first arise from hemogenic endothelium in the dorsal aorta (Bertrand *et al*, 2010; Mahony *et al*, 2021). Through blood circulation, they colonize a transient hematopoietic niche before they settle in the adult marrow. In mammals, this temporary niche is the fetal liver (FL), whereas the caudal hematopoietic tissue (CHT) plays this role in zebrafish embryos (Tamplin *et al*, 2015). This transitory niche is the only one where HSCs will expand extensively, up to 40 times in the mouse embryo (Ema & Nakauchi, 2000). Using the zebrafish embryo, we and others have discovered many signaling pathways involved in HSC expansion (Tamplin *et al*, 2015; Cacialli *et al*, 2021). Among these molecules, the prostaglandin E (PGE2) has shown promising results both experimentally and clinically (Goessling *et al*, 2011), although it was originally described as an enhancer of HSC specification from the hemogenic endothelium (North *et al*, 2007).

Prostaglandin E_2_ is the most abundant prostaglandin in the organism. It is synthesized through the sequential oxygenation of arachidonic acid (AA). AA derives from diacylglycerol or phospholipids, present in the cell membrane, that are oxidized by phospholipases (from the C or A2 family). AA is then metabolized by the cyclooxygenases *cox1* and *cox2* to produce PGG2/PGH2, before the *Prostaglandin E‐synthase* (*ptges*), an isomerase, synthetises PGE2. Many studies showed that PGE2 enhances HSC specification and/or proliferation and that blocking prostaglandin synthesis decreases HSC numbers (North *et al*, 2007). PGE2 can bind to four different G‐coupled receptors, EP 1–4, which have various cellular functions (Sugimoto & Narumiya, 2007). Both EP2 and EP4 receptors are expressed by hematopoietic precursors (North *et al*, 2007), and transduce PGE2 signaling through the cAMP/PKA pathway. EP4 additionally activates the phosphatidylinositol 3‐kinase (PI3K)/AKT pathway (Fujino *et al*, 2003). However, the precise cellular source of PGE2 in the embryo has yet to be cleared. Here, we show that all the genes involved in PGE2 synthesis are expressed by different cells of the CHT in the embryonic zebrafish, a pattern that seems conserved also in the mouse FL. In the zebrafish CHT, as in mouse FL, we find that neutrophils express high levels of phospholipases, while macrophages express *cox1*/*2* enzymes and endothelial cells (ECs) high levels of *ptges*. This suggests that each cell type is sequentially necessary to mediate PGE2 synthesis. To measure the impact of myeloid cells, we generated a genetic model of myeloid ablation, which caused a loss of HSCs in the CHT, and could be rescued by the addition of PGE2 or PGH2. Moreover, we identified the role of an important transporter, *slco2b1*, that mediates the transport of prostaglandins into ECs. This transporter is a member of the Organic Anion Transporting Polypeptides (OATP) superfamily, which is largely conserved between zebrafish and mammals (Popovic *et al*, 2010). Indeed, all zebrafish OATPS consists of 12 transmembrane domains with a larger fifth extracellular loop, LP5, that contains 10 conserved cysteine residues. These conserved cysteine residues within LP5 are found to be crucial for protein function (Hanggi *et al*, 2006). In the present report, we found a defect of HSC expansion in the CHT of *slco2b1*‐deficient embryos, which could also be rescued by exogenous PGE2. Taken altogether, our data show that the myeloid cells and the vascular niche cooperate to enhance HSC expansion in the CHT.

## Results

### The PGE2 synthesis pathway is distributed over three different cell types in the CHT

**Figure 1:**
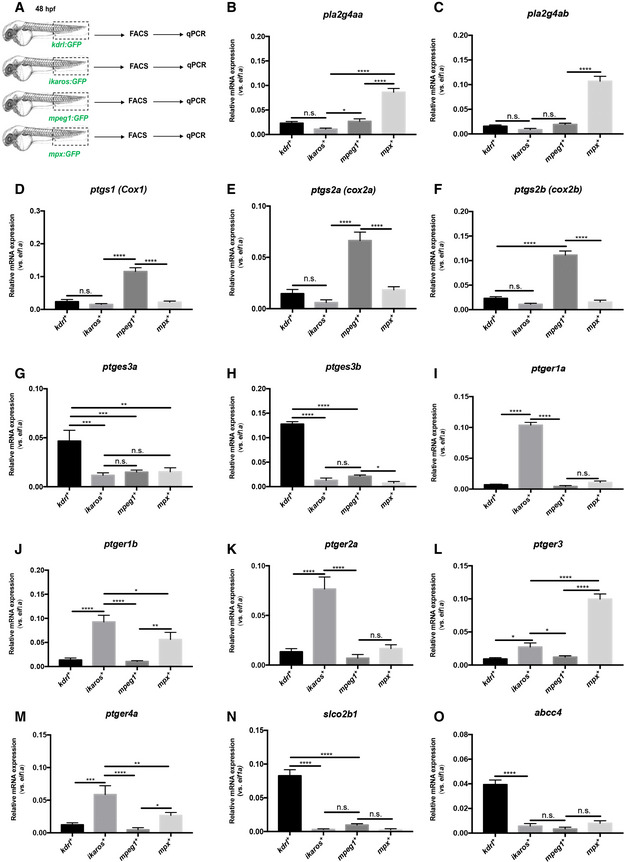
The PGE_2_ synthesis pathway in zebrafish CHT A
Experimental outline of qPCR analysis after dissection of CHT (caudal region) at 48 hpf from *kdrl:GFP*, *ikaros:GFP*, *mpeg1:GFP*, *mpx:GFP* transgenic animals, and FACS‐sorted GFP‐positive cells to purify ECs, HSPCs, macrophages, and neutrophils.B, C
The phospholipases *pla2g4aa* and *pla2g4ab* are highly expressed in neutrophils.D–F
The expression of cyclooxygenases *cox1*, *cox2a*, *and cox2b* is enriched in macrophages.G, H
The prostaglandin synthases *ptges3a* and *ptges3b* are only expressed by ECs.I–M
The prostaglandin receptors *ptger1a*, *ptger1b*, *ptger2a*, *ptger3*, and *ptger4a* are mostly expressed in HSPCs.N, O
The prostaglandin transporters *slco2b1* and *abcc4* are specifically expressed in ECs. Data information: For each panel, data represent biological triplicates plated in technical duplicates. Statistical analysis was completed using one‐way ANOVA and multiple comparison tests. *P < 0.05; **P < 0.01; ***P < 0.001; ****P < 0.0001. Center values denote the mean, and error bars denote s.e.m. Source data are available online for this figure.

In order to accurately describe the PGE2 synthesis pathway, we quantified the expression of the genes coding for the enzymes, channels, and receptors involved in PGE2 signaling, in different populations of the zebrafish CHT. We dissected CHTs (caudal regions) at 48 hpf from *kdrl:GFP*, *ikaros:GFP*, *mpeg1:GFP*, and *mpx:GFP* transgenic animals to purify ECs, HSPCs, macrophages, and neutrophils, respectively (Fig 1A). By qPCR analysis, we find that the phospholipases *pla2g4aa* and *pla2g4ab* are highly expressed in neutrophils (Fig 1B and C), while the expression of cyclooxygenases *cox1*, *cox2a*, and *cox2b* is enriched in macrophages (Fig 1D–F). Finally, we find that the prostaglandin synthases *ptges3a* and *ptges3b* are highly expressed in ECs (Fig 1G and H). As expected, and as previously reported (North *et al*, 2007) the prostaglandin receptors *ptger1a*, *ptger1b*, *ptger2a*, and *ptger4a* are mostly expressed in HSPCs. *ptger3* and *ptger4a*, to a lesser extent, were expressed in neutrophils (Fig 1I–M). These data establish that in the CHT, different cell types may play an important role in PGE2 production. PGE2 release and uptake are required to initiate and terminate their biological actions, respectively. Thus, efficient transport of PGE2 across the cell membrane is critical. This can be mediated by the transmembrane transporters ATP‐binding cassette, subfamily C, member 4 (*abcc4*; also known as *MRP4*), and solute carrier organic anion transporter (OATP) family members, such as *SLCO2B1*. While *ABCC4* plays a role in PGE2 release from the producing cell (efflux PGE2 transporter; Russel *et al*, 2008), *SLCO2B1* promotes PGE2 uptake from the extracellular space (influx PGE2 transporter) (International Transporter Consortium *et al*, 2010) but could also contribute to the uptake of other metabolites of the prostaglandin family (Li *et al*, 2020). Previous studies in mammals showed that *slco2b1* and *abcc4* are highly expressed in hepatocytes in the adult liver but also in other tissues including the intestines, heart, and brain capillary ECs (Tamai *et al*, 2000). In the zebrafish CHT, we find that *slco2b1* and *abcc4* are highly expressed in caudal ECs during development, as determined by qPCR (Fig 1N and O), and confirmed by *in situ* hybridization at different stages of embryo development, and we confirmed by double WISH that *slco2b1* and *flk1* expressions overlapped in the ventral part of the CHT (Appendix Fig S1A–C). Altogether, our data suggest that PGE2 synthesis is achieved by many cell subsets in the hematopoietic niche.

### The specific ablation of myeloid cell decreases the number of HSCs in the CHT

**Figure 2:**
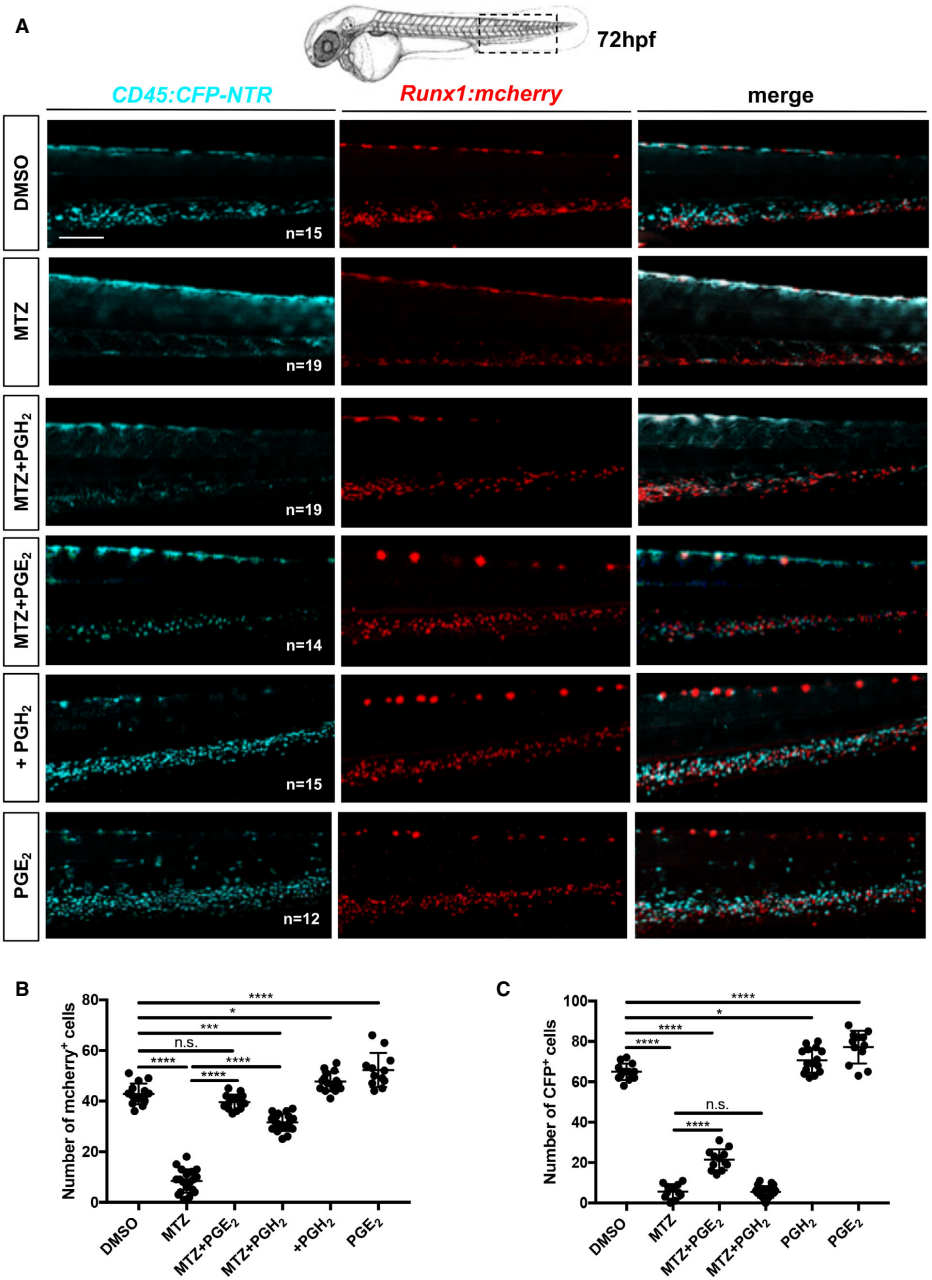
The loss of HSCs after myeloid ablation can be rescued by PGE2 or PGH2 treatments A
Schematic indicating the imaging area in the tail at 72 hpf, as indicated by the black box; fluorescence imaging of the CHT in double transgenic *cd45:CFP‐NTR*/*runx1:mcherry* embryos in DMSO and after treatment with MTZ and PGH2 or PGE2_._
B
Quantification of *runx1:mcherry*‐positive cells in double transgenic *cd45:CFP‐NTR*/*runx1:mcherry* embryos in DMSO and after treatment with MTZ and PGH2 or PGE2_._
C
Quantification of *cd45:CFP‐*positive cells in double transgenic *cd45:CFP‐NTR*/*runx1:mcherry* embryos in DMSO and after treatment with MTZ and PGH2 or PGE2_._ Data information: Statistical analysis was completed using one‐way ANOVA and multiple comparison tests. *P < 0.05; **P < 0.01; ***P < 0.001; ****P < 0.0001. Scale bar is 200 μm (A). Source data are available online for this figure.

During zebrafish development, HSCs have been shown to mainly interact with ECs and other cells in the CHT. However, myeloid cells could have an important role as they express the key enzymes to initiate PGE2 production. To investigate whether and how myeloid cells contribute to HSCs expansion, we genetically ablated these cell types during development. This was achieved using a *Tg*(*cd45:CFP‐NTR*) zebrafish line in which the bacterial nitroreductase (NTR), fused to CFP, is expressed in *ptprc(cd45)*‐positive cells (Appendix Fig S2A). Although CD45 is a pan‐leukocytic marker, we have previously shown that the 7.6 kb promoter we isolated is only transcriptionally active in myeloid and T cells (Wittamer *et al*, 2011) but not in hematopoietic progenitors or any other hematopoietic subsets in adult zebrafish. To assess this during embryogenesis, we analyzed triple transgenic *cd45:CFP‐NTR;mpeg1:mCherry;mpx:GFP* embryos at 60hpf by live imaging (Appendix Fig S2B). Our observations indicate that all mCherry and/or GFP‐positive cells also expressed CFP, confirming that our *cd45* promoter is active in macrophage and neutrophil cells. We did not observe any single CFP‐positive cells, indicating that at this stage, the *cd45* promoter is only active in myeloid cells. Next, we analyzed double transgenic *cd45:CFP‐NTR*;*runx1:mCherry* embryos at 48 and 72 hpf by flow cytometry, and triple transgenic *cd45:CFP‐NTR*;*runx1:mCherry;mpeg1:GFP* embryos at 60 hpf by live imaging (Appendix Fig S3A–C). Our data clearly show that CFP and mCherry never overlap, meaning that the 7.6 kb *cd45* promoter is inactive in embryonic HSPCs. As previously reported, the ablation system allows the killing of NTR‐expressing cells upon the addition of metronidazole (MTZ) (Pisharath, 2007). Importantly, expression alone or administration of MTZ to nontransgenic embryos does not induce apoptosis. By contrast, a single treatment with MTZ between 48 and 72 hpf was sufficient to completely ablate myeloid cells in *cd45:CFP‐NTR* transgenic embryos, which was confirmed by performing *in situ* hybridization for *mfap4* and *mpx* (Appendix Fig S4A and B). Since our previous results suggested that myeloid cells have a relevant role in PGE2 synthesis in the CHT, we treated *cd45:CFP‐NTR* transgenic embryos with DMSO and/or MTZ (between 48 and 72 hpf) (Appendix Fig S5A), and found a significative decrease in PGE2 levels after myeloid ablation, measured by ELISA (Appendix Fig S5B), according to the manufacturer's protocols and as previously reported (Esain *et al*, 2015). This could be explained by the absence of phospholipases and *cox1*/*2* enzymes after myeloid ablation, as scored by qPCR (Appendix Fig S6A–F). Interestingly, after myeloid ablation, we found no change in the expression of *slco2b1*, *abcc4*, and *ptges3a* (Appendix Fig S6G–I), and a small decrease in *ptges3b* (Appendix Fig S6L). Finally, we find that myeloid ablation decreases the expression of prostaglandin receptors *ptger1a*, *ptger1b*, *ptger2a*, *ptger3*, and *ptger4a* (Appendix Fig S6M–Q), which could be explained by the expression of these receptors by some myeloid subsets (Fig 1) but also probably by the impact on HSC expansion. These results confirm that myeloid cells have an important role in the production of PGE2 in the CHT niche, and that their absence cannot be compensated by other cells in the hematopoietic niche. Next, we treated *cd45:CFP‐NTR;runx1:mCherry* double transgenic embryos between 48 and 72 hpf with DMSO and/or MTZ. As expected, the number of *runx1:mCherry* ^+^ cells decreased in the CHT of double transgenic embryos at 72 hpf, compared with controls (Fig 2A and B). MTZ treatment on nontransgenic embryos did not affect HSCs at any tested concentrations (1–5‐10 mM), indicating that our phenotype was specifically due to myeloid ablation (Appendix Fig S7A). We could rescue this phenotype by supplementing MTZ‐treated embryos with PGE2 or PGH2 (Fig 2A and B), but treatments with AA or PGG2 did not rescue the numbers of *runx1*‐positive cells and or myeloid cells (CFP^+^) in the CHT (Appendix Fig S8A–C). We also quantified the number of *cd45*‐positive cells and only PGE2 treatment could modestly rescue the number of myeloid cells after ablation (Fig 2C). In summary, our data indicate that myeloid cells are necessary for PGE2 synthesis, as they ultimately contribute to the production of PGH2, which will then need to be metabolized into PGE2 to promote HSC expansion.

### *slco2b1*‐deficient embryos show a severe decrease in HSC expansion in the CHT

**Figure 3:**
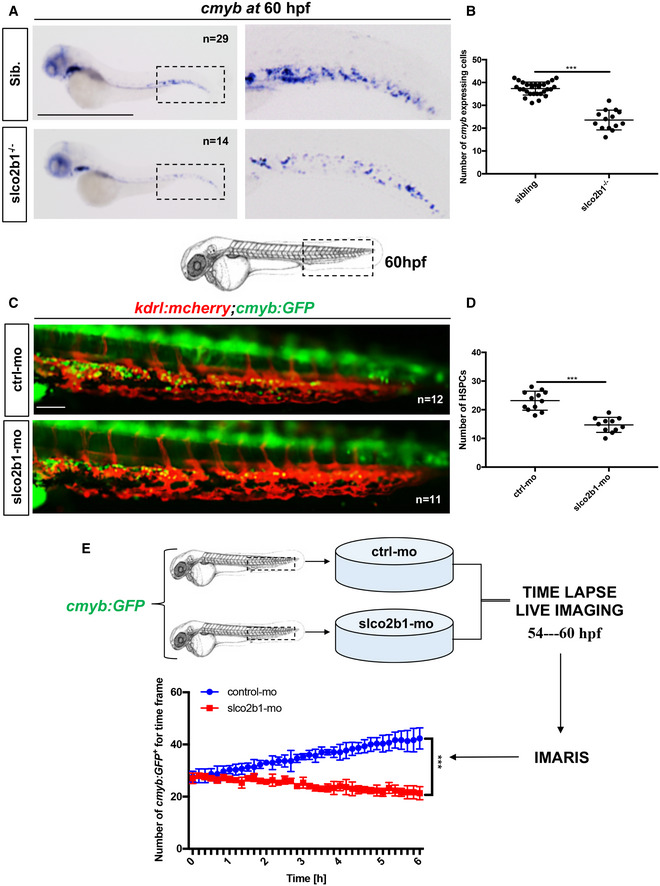
The deficiency of *slco2b1* induces a decrease of HSPC in the CHT A
WISH for *cmyb* expression at 60 hpf in wild‐type and *slco2b1* ^−/−^ embryos.B
Quantification of *cmyb‐*expressing cells. Each *n* represents the number of *cmyb*‐expressing cells for each embryo (biological replicates). Each experiment has been repeated three independent times.C
Schematic indicating the imaging area in the tail at 60 hpf, as indicated by the black box; fluorescence imaging in the CHT of *kdrl:mCherry;cmyb:GFP* embryos injected with control‐ and *slco2b1*‐Mos.D
Quantification of HSPCs associated with ECs. Each *n* represents the number of yellow spots for each embryo (biological replicates). Each experiment has been repeated three independent times.E
Experimental outline and quantification of time‐lapse live imaging in controls and *slco2b1*‐morphants *cmyb:GFP* ^+^ cells, using Imaris software. Data information: Center values denote the mean, and error values denote s.e.m. The statistical analysis was completed using an unpaired two‐tailed *t*‐test. ***P < 0.001. Scale bar is 500 μm (A); 200 μm (C). Source data are available online for this figure.

**Figure 4:**
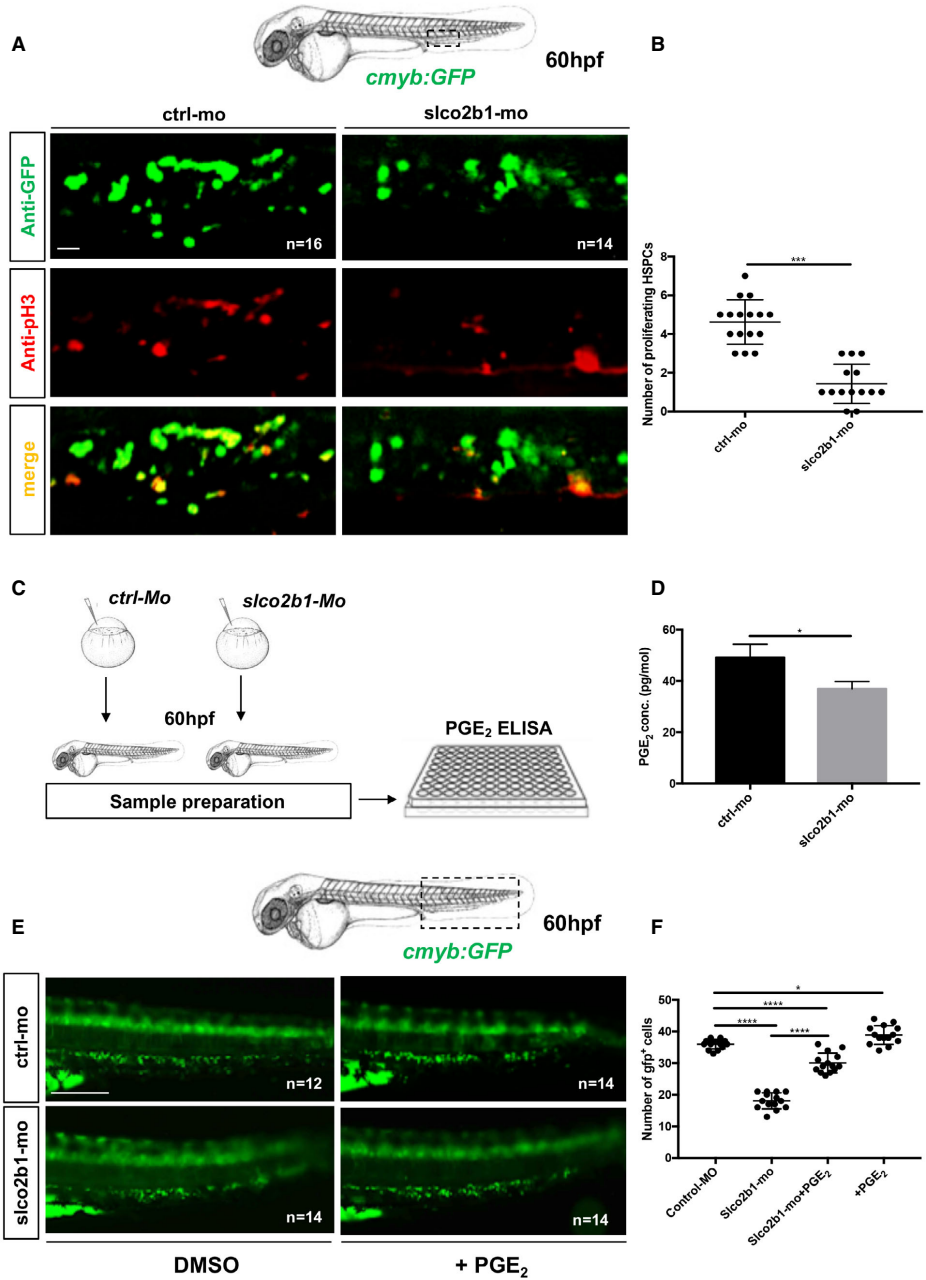
The defect in HSPCs proliferation in *slco2b1*‐deficient embryos can be rescued by PGE2 treatment A
Anti‐GFP and pH3 immunostainings of either controls (Ctrl‐Mo) or *slco2b1*‐morphants (*slco2b1‐MO*) *cmyb:GFP* embryos.B
Quantification of the number of the pH3+ HSCs in controls or *slco2b1*‐morphants. Center values denote the mean, and error values denote s.e.m, statistical analysis was completed using an unpaired two‐tailed *t*‐test. ***P < 0.001.C
Experimental outline to measure PGE2 level by ELISA kit in control and *slco2b1‐MO* at 60 hpf.D
Quantification of PGE2 concentration in control‐ and *slco2b1‐*morphants. The statistical analysis was completed using an unpaired two‐tailed *t*‐test *P < 0.01. Center values denote the mean, and error bars denote s.e.m.E
Fluorescence imaging in the CHT of *cmyb:GFP* embryos injected with control‐ and *slco2b1*‐MOs and treated with PGE_2_.F
Quantification of *GFP*‐positive cells. Statistical analysis: one‐way ANOVA, multiple comparison, *P < 0.01; ****P < 0.0001. Center values denote the mean, and error bars denote s.e.m. Data information: Scale bar is 50 μm (A); 200 μm (E). Source data are available online for this figure.

As mentioned before, myeloid cells can produce PGG2/PGH2 from AA but are unable to convert this precursor into PGE2 as they lack the expression of *ptges* enzymes, which are highly expressed in caudal ECs. Therefore, PGG2/PGH2 has to be transferred to ECs to be processed. OATP channels are specialized channels involved in intracellular influx (International Transporter Consortium *et al*, 2010). In particular, *SLCO2B1* has been shown to promote the entry of PGE2 or similar metabolites into different cell types. As *slco2b1* expression is highly enriched in ECs of the CHT, we then investigated the consequences of *slco2b1*‐deficiency on HSCs during zebrafish embryo development. We used the uncharacterized *slco2b1* ^*sa37367*^ mutant line, which presents a point mutation in the splice donor site at the end of exon 4 (Appendix Fig S9A). In this mutant, the transcript for slco2b1 was completely undetectable (Appendix Fig S9B). By *in situ* hybridization, we show that *slco2b1* ^−/−^ embryos exhibit a defect in definitive hematopoiesis as early as 60hpf (Fig 3A and B), which was maintained at 4 dpf (Appendix Fig S9C). However, no change in *runx1* and *cmyb* expression were detected at 28 and 36 hpf, respectively, showing that *slco2b1* is not involved in HSC specification from the hemogenic endothelium (Appendix Fig S9D and E). Of note, *slco2b1*‐deficient embryos did not show any changes in the development of early vasculature or in primitive hematopoiesis (Appendix Fig S10A–C), showing an exclusive role for *slco2b1* during HSPC expansion in the CHT. Morpholino‐mediated knockdown of *slco2b1* expression completely phenocopied *slco2b1* ^−/−^ mutant embryos, where HSC specification was unaffected, but their expansion impaired in the CHT (Appendix Fig S11A–C). To confirm our results, we first injected control‐ and *slco2b1*‐morpholinos in *kdrl:mcherry;cmyb:GFP* embryos and scored the number of HSCs at 60hpf, where we observed a significant decrease in double‐positive cells in *slco2b1*‐morphants (Fig 3C and D). To determine whether HSCs underwent cell death in *slco2b1*‐deficient animals, we performed an apoptosis assay by acridine orange staining on control‐ and *slco2b1*‐morphants, but we did not observe any significative difference (Appendix Fig S12A and B). Next, we used time‐lapse confocal imaging to follow the HSCs' behavior in the CHT in *cmyb:GFP slco2b1‐*morphants. While the number of HSCs augmented in control embryos between 54 and 60 hpf, their number remained unchanged in *slco2b1*‐deficient embryos (Fig 3E; Movies EV1 and EV2). These results suggested that the absence of *slco2b1* in the vascular niche generated a defect of HSC proliferation in the CHT. To confirm this hypothesis, we injected control‐ and *slco2b1*‐morpholinos in *cmyb:GFP* embryos and stained for both GFP and phospho‐Histone 3 (pH3) to quantify proliferating HSCs. At 60hpf, *slco2b1*‐morphants showed a significant decrease in the number of proliferating HSCs (Fig 4A and B). Therefore, the function of *slco2b1* in ECs is necessary for the expansion of HSCs in the CHT.

### PGE2, but not PGH2, rescues the loss of HSCs observed in *slco2b1*‐deficient embryos

**Figure 5:**
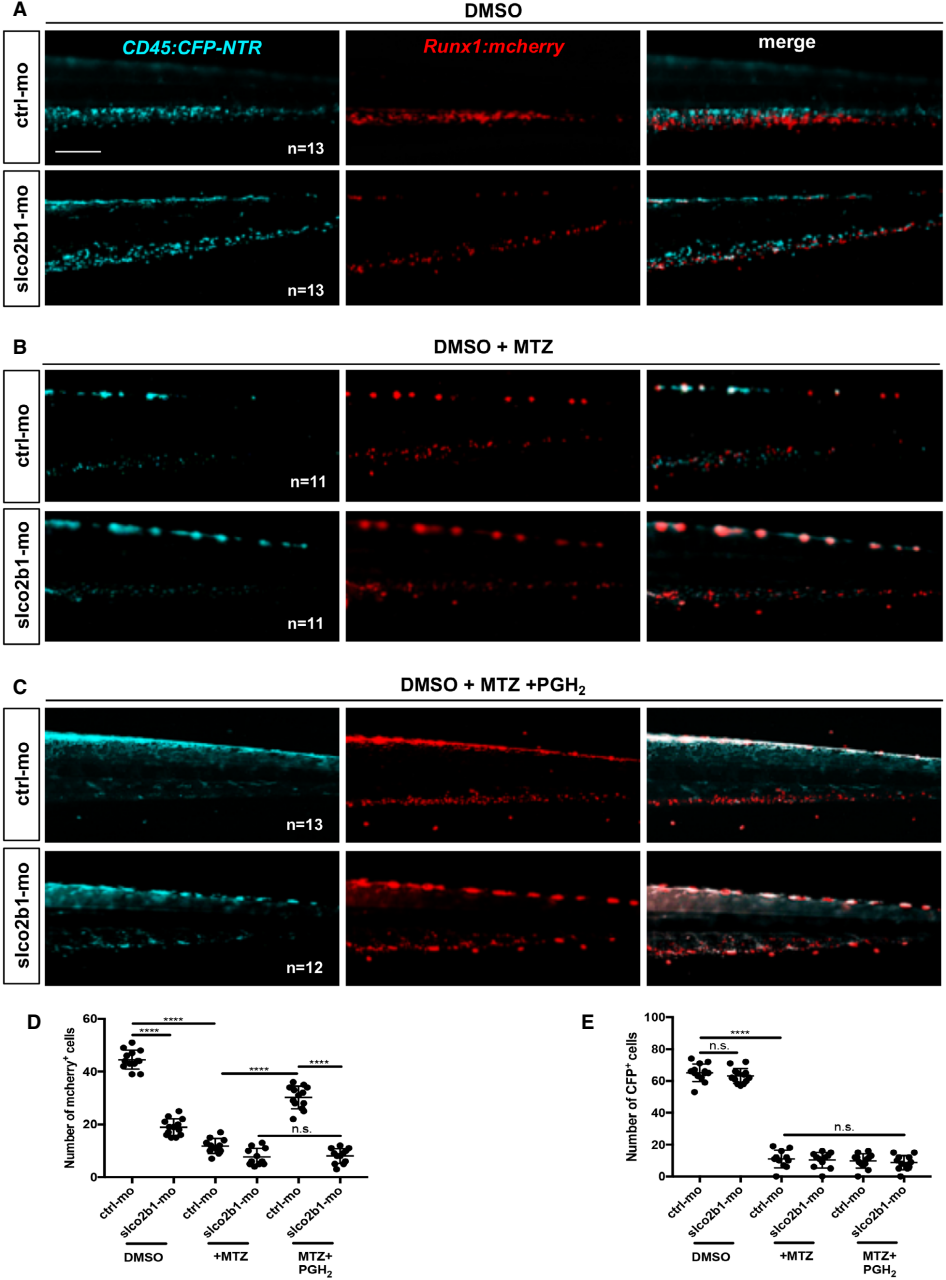
*Slco2b1* is necessary to import PGH2 into ECs A
Fluorescence imaging of the CHT of double transgenic *cd45:CFP‐NTR*/*runx1:mcherry* embryos injected with control‐ or *slco2b1*‐morpholinos, after treatment with DMSO.B
Fluorescence imaging of the CHT of double transgenic *cd45:CFP‐NTR*/*runx1:mcherry* embryos injected with control‐ or *slco2b1*‐morpholinos, after treatment with DMSO+MTZ_._
C
Fluorescence imaging of the CHT of double transgenic *cd45:CFP‐NTR*/*runx1:mcherry* embryos injected with control‐ or *slco2b1*‐morpholinos, after treatment with DMSO+MTZ + PGH2.D
Quantification of *runx1:mcherry*‐positive cells. Each dot represents the number of red cells for each embryo (biological replicates). Each experiment has been repeated three independent times.E
Quantification of *cd45:CFP‐*positive cells_._ Each dot represents the number of blue cells for each embryo (biological replicates). Each experiment has been repeated three independent times. Data information for (D) and (E): Statistical analysis was completed using one‐way ANOVA and multiple comparison tests. ****P < 0.0001. Center values denote the mean, and error bars denote s.e.m. Scale bar is 200 μm (A–C). Source data are available online for this figure.

**Figure 6:**
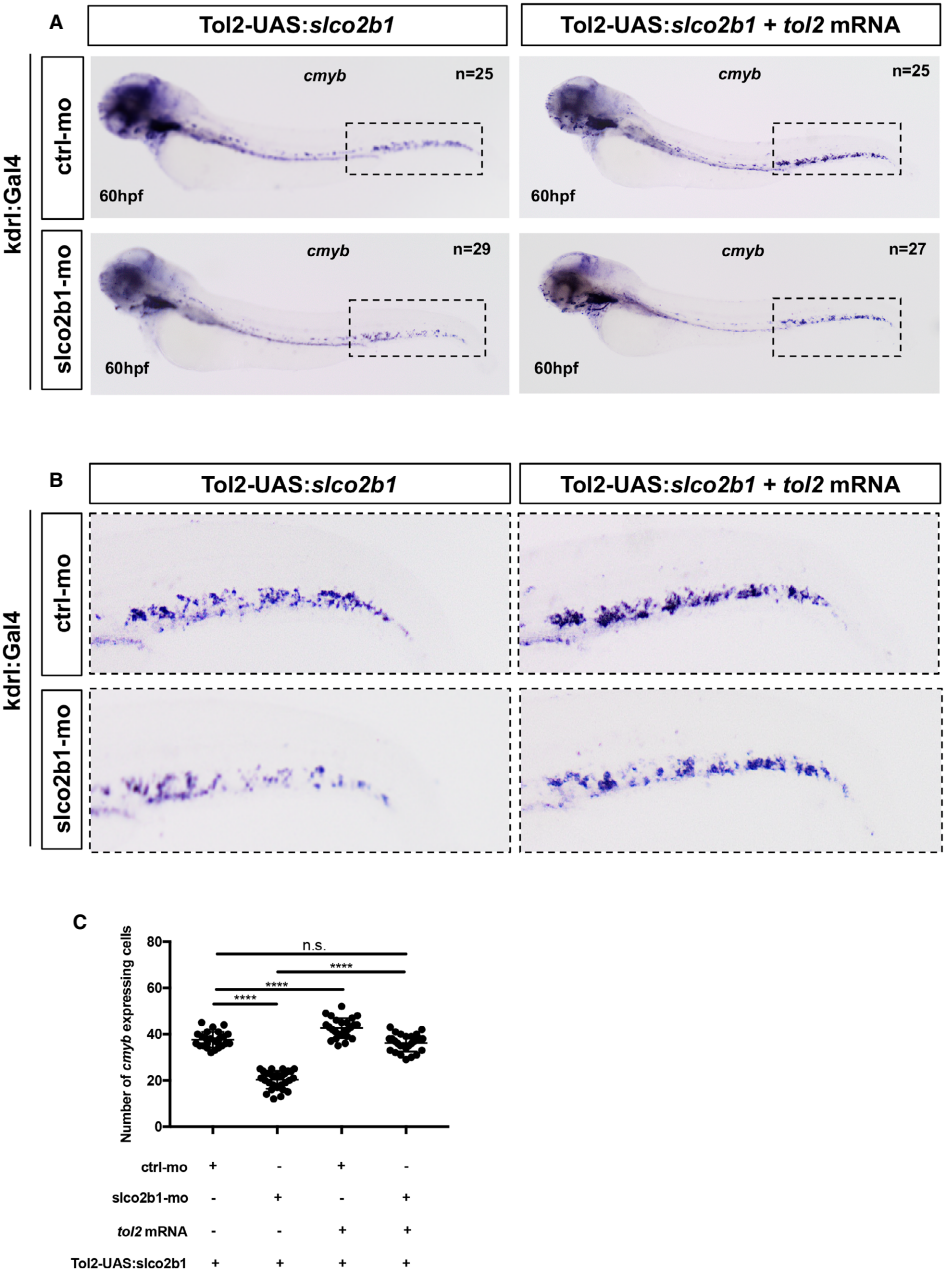
*Slco2b1*‐overexpression in ECs can rescue the loss of HSPCs in *slco2b1*‐ morphants A,B
(A) WISH for *cmyb* at 60hpf in *kdrl:Gal4*+ embryos injected with control‐ and *slco2b1*‐ morpholinos and/or co‐injected with the *Tol2‐UAS:slco2b1* vector and *tol2* mRNA. The region indicated by the dashed has been magnified and shown in (B).C
Quantification and statistical analysis were completed using one‐way ANOVA and multiple comparison tests. ****P < 0.0001. Center values denote the mean, and error bars denote s.e.m. Each dot represents the number of *cmyb*‐expressing cells for each embryo (biological replicates). Each experiment has been repeated three independent times. Source data are available online for this figure.

Based on our results, the deficiency of *slco2b1* induces a defect of HSC proliferation, probably caused by a decrease in PGE2 in the CHT of the zebrafish embryo. However, as mentioned earlier, *slco2b1* is a transporter used for the uptake of PGE2, and other prostaglandins, therefore the absence of *slco2b1* could potentially result in an increase in PGE2 in the extracellular environment. In order to evaluate PGE2 levels in *slco2b1*‐morphants, we injected wild‐type AB* embryos with control‐ and *slco2b1‐*morpholinos, and at 60hpf we processed embryos and measured PGE2 levels by ELISA assay (Fig 4C and D). We found a significant decrease in PGE2 levels in *slco2b1*‐morphants compared with controls (Fig 4D), suggesting that less PGE2 is produced in these embryos, concordant with the defect observed in HSCs. Therefore, we investigated the role of *slco2b1* in PGE2 synthesis. First, we injected AB* embryos with control‐ and *slco2b1*‐morpholinos and supplemented them with PGE2, PGH2, PGG2 or AA, from 48 to 60 hpf. We then anlaysed *cmyb* expression by WISH and counted the number of *cmyb‐*expressing cells in the CHT at 60 hpf (Appendix Fig S13A). We found that only PGE2 treatment rescued the loss of HSCs in *slco2b1*‐morphants (Appendix Fig S13B). This was confirmed by live imaging using the *cmyb:GFP* transgenic reporter (Fig 4E and F), and we also confirmed that HSCs numbers where similarly rescued in *slco2b1* mutant embryos (Appendix Fig S13C and D). As PGH2 (normally produced by macrophages) could not rescue the *slco2b1* deficiency, we hypothesized that ECs could import PGH2 through this transporter. To verify this, we injected *cd45:CFP‐NTR*;*runx1:mCherry* embryos with control‐ and *slco2b1*‐morpholinos and we treated them with DMSO, MTZ, and/or PGH2, from 48 to 72 hpf, and counted the number of *mCherry* ^+^ cells at 72 hpf for all conditions. As expected, *slco2b1*‐morphants treated with DMSO showed less HSCs (mCherry^+^) than control embryos (Fig 5A–D). Control‐ and *slco2b1*‐morphants treated with MTZ, where myeloid cells (CFP^+^) were ablated, showed a strong decrease in HSCs (Fig 5B–D). Interestingly, control‐morphants treated with MTZ and supplemented with PGH2 showed fairly normal numbers of HSPCs, despite the ablation of most myeloid cells, but MTZ‐treated *slco2b1*‐morphants were not rescued by PGH2 (Fig 5C and D). We also quantified the number of *cd45*‐positive cells and PGH2 could not rescue the number of myeloid cells after ablation (Fig 5E). These data confirm the hypothesis that in the absence of *slco2b1* transporter, caudal ECs cannot import the macrophage‐derived PGH2, a metabolite necessary to produce PGE2. Taken all together, our data suggest that the function of *slco2b1* in ECs is necessary for the expansion of HSCs. In order to investigate the specific role of *slco2b1* in ECs, we overexpressed *slco2b1* transiently by injecting the Tol2‐UAS:*slco2b1* construct together with *tol2* mRNA in either *kdrl:Gal4* or *mpeg1:Gal4* embryos, and we performed WISH for *slco2b1* to confirm integration and tissue‐specific overexpression (Appendix Fig S14A and B). Next, to evaluate the impact of *slco2b1*‐overexpression in ECs or macrophages on HSCs, we scored *cmyb* at 60hpf in Gal4‐reporter embryos injected with control‐ or *slco2b1*‐morpholinos and/or co‐injected with Tol2‐UAS:*slco2b1* and *tol2* mRNA. As the *slco2b1*‐morpholino targets a splice junction, this experiment allowed us to selectively rescue *slco2b1* expression in ECs or macrophages when the endogenous expression of *slco2b1* was abolished in all cell types. We found that the deficiency of HSCs in *slco2b1*‐morphants can be rescued only when *slco2b1* expression was restored into caudal ECs (Fig 6A–C) but not in macrophages (Appendix Fig S15A–C). These results confirm that *slco2b1* has a specific role in CHT‐ECs by promoting the import of macrophage‐derived PGH2 to synthetize PGE2.

### Mouse fetal liver expression levels of PGE2 synthetic pathway

**Figure 7:**
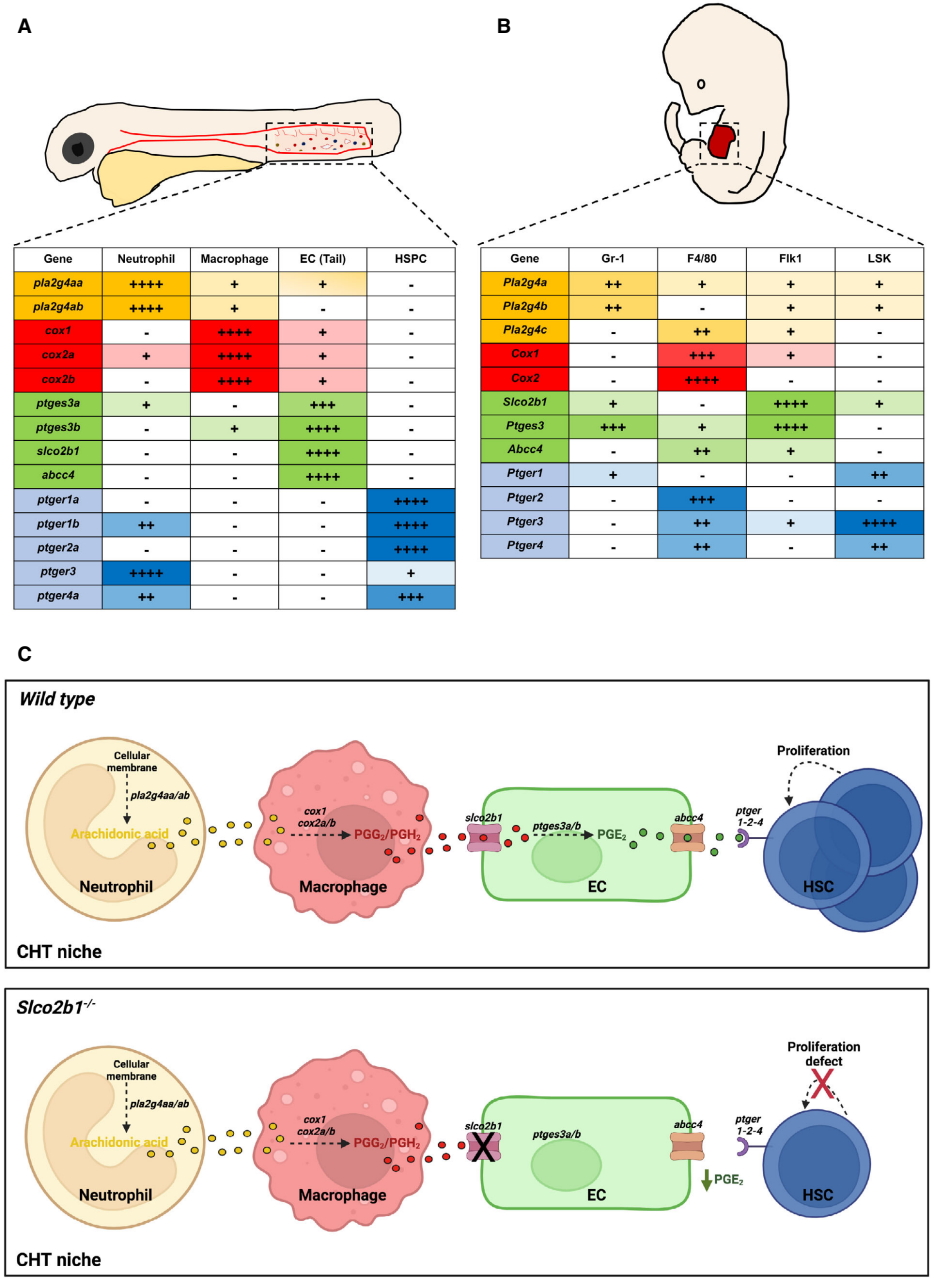
The expression of the prostaglandin synthesis pathway is conserved between the zebrafish CHT and the mouse fetal liver A, B
(A) Summary of prostaglandin gene expression pathways in the CHT of zebrafish, and (B) in mouse FL. The different symbols correspond to the *C*_t_ value to which the threshold of detection was applied during the analysis of the qPCR: very high expression (++++) *C*_t_ < 24; high expression (+++) 25 < *C*_t_ < 28; medium expression (++) 29 < *C*_t_ < 32; low expression (+) 33 < *C*_t_ < 36; very low to no expression (−) *C*_t_ > 37.C
Proposal mechanism in which HSCs, ECs, macrophages and neutrophils cooperate in the embryonic niche. In normal conditions (upper panel) *slco2b1* permits the transfer of PGE2 precursors in ECs. The deficiency of *slco2b1* (lower panel) decreases PGE2 levels in the CHT niche, generating a defect of HSC proliferation. Source data are available online for this figure.

**Figure 8:**
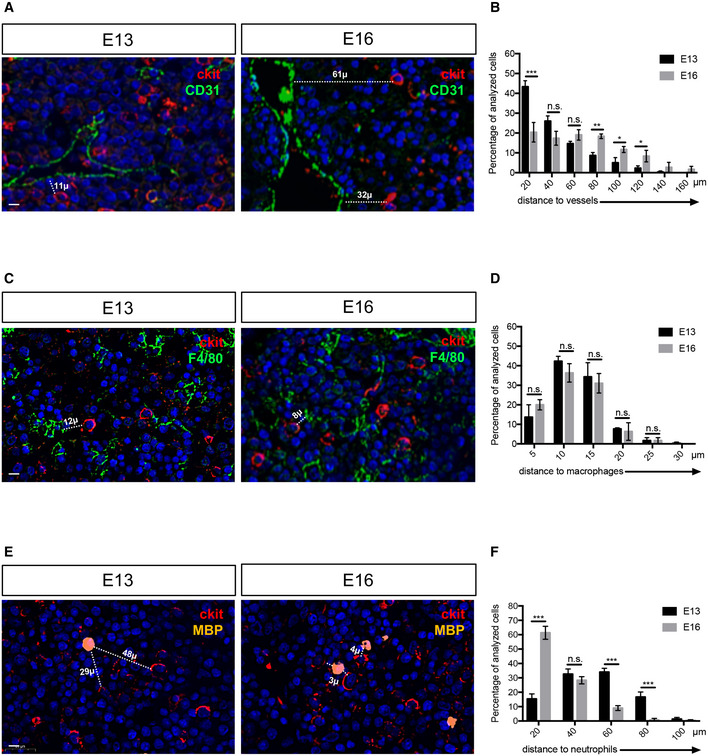
Myeloid and endothelial cells are very close to HSPCs in the mouse fetal liver A
Immunohistochemistry on paraffin sections for cKit (HSPCs marker) and CD31 (endothelial cell marker) at E13 and E16 stages of mouse fetal liver.B
Distance distribution between cKit^high^ cells and endothelial cells (*n* = 79 from three E13 fetal livers; *n* = 92 from three E16 fetal livers), binned into 20 μm intervals. Statistical analysis was completed using a *t*‐test. *P < 0.01; **P < 0.001; ***P < 0.0001. Center values denote the mean, and error values denote s.e.m.C
Immunohistochemistry on paraffin sections for cKit (HSPCs marker) and F4/80 (macrophage cell marker) at E13 and E16 stages of mouse fetal liver.D
Distance distribution between cKit^high^ cells and macrophages (*n* = 54 from three E13 fetal livers; *n* = 64 from three E16 fetal livers), binned into 5 μm intervals. Statistical analysis was completed using a *t*‐test.E
Immunohistochemistry on paraffin sections for cKit (HSPCs marker) and MBP (neutrophil cell marker) at E13 and E16 stages of mouse fetal liver.F
Distance distribution between cKit^high^ cells and neutrophils (*n* = 45 from three E13 fetal livers; *n* = 53 from three E16 fetal livers), binned into 20 μm intervals. Statistical analysis was completed using a *t*‐test ***P < 0.001. Data information: All nuclei were marked with DAPI. Scale bar is 20 μm (A–C–E).

In order to determine whether the prostaglandin pathway present in the zebrafish CHT was also conserved in mammals, we performed qPCR on FACS‐sorted cell subsets from mouse FL at E14.5 by using various combinations of antibodies. HSCs were isolated based on their Lin^−^Sca1^+^cKit^+^ (LSK) phenotype; F4/80 also known as EMR1 or Ly71, was used as a macrophage marker, *Flk1* and CD31 to mark ECs and Gr‐1, known as Ly‐6G/Ly‐6C, to sort neutrophils. By qPCR, we found that the phospholipase *Pla2g4a* was highly expressed in neutrophils (Appendix Fig S16A), while *Pla2g4b* and *Pla2g4c* were expressed at very low levels (Appendix Fig S16B and C). As in the zebrafish, *Cox1* and *Cox2* were mostly expressed in macrophages (Appendix Fig S16D and E). We also found a high enrichment of *Ptges3*, *Slco2b1*, and *Abcc4* in ECs (Appendix Fig S16F–H). Next, we examined the expression of the prostaglandin receptors. *Ptger1*, *Ptger3*, and *Ptger4* were mostly expressed in HSCs, and *Ptger2* was expressed in macrophages (Appendix Fig S16I–N). By comparing zebrafish and mouse patterns of expression, we confirm that the distribution of the prostaglandin pathway has been conserved between the zebrafish CHT and the mouse FL (Fig 7A and B). Based on these results, we propose a model in which several cell types in the CHT niche, and in the mouse FL, concur together to the production of PGE2 and therefore to the expansion of HSCs (Fig 7C upper panel). However, in the absence of *slco2b1*, the PGE2 biosynthetic chain is interrupted and HSCs cannot expand (Fig 7C lower panel). To support our model, and verify if the cells are in close proximity in mouse FL at E13 and E16, we performed immunohistochemistry using different cell markers: cKit to mark HSPCs; CD31 to mark endothelial cells; F4/80 to mark macrophages and MBP to mark neutrophils. Using a digital microscopy application (CaseViewer‐3DHistech) we measured distances between HSPCs (cKit^hi^) and other cell types of interest: endothelial cells, macrophages, and neutrophils. As reported in previous studies (Khan *et al*, 2016), we found that HSPCs stand very close to the vasculature at E13 (Fig 8A). HSPCs' distance to vessels increased significantly during mouse fetal liver development (Fig 8B). As for macrophages, we could observe that HSPCs were always in their close vicinity at both stages (Fig 8C and D). Finally, we found that the HSPC distances to neutrophils decreased during development (Fig 8E). Indeed, at E16 all HSPCs were very close to neutrophils (60% of cells <20 μm), which could also be explained by the overall increase in neutrophil numbers through development (Fig 8F). In summary, at E13, HSPCs were closely associated with endothelial cells and macrophages.

## Discussion

Prostaglandin E_2_ plays important roles during inflammation. However, these effects can be opposite depending on the cell type targeted. Indeed, different immune cell types will express different PGE2 receptors. Indeed, many studies have shown that PGE2 not only stimulates the specification of HSCs from the hemogenic endothelium (North *et al*, 2007) but also their expansion, which has been successfully translated to nonhuman primates and patients (Goessling *et al*, 2011). The mammalian FL, or the CHT in the zebrafish embryo, are thought to be the only niches where HSCs actively proliferate. Therefore, we hypothesized that the PGE2 synthesis pathway was active in this microenvironment. Surprisingly, we found that all the key enzymes necessary for PGE2 synthesis (phospholipases, *cox1*/*2*, and prostaglandin E‐synthase) are distributed between different cell types: neutrophils, macrophages, and ECs. This finding involves a close collaboration between these cell types and supposes that metabolites can transit from one cell type to another. The intercellular collaboration we show here is not an isolated example. Indeed, many other molecules are produced according to similar mechanisms, in multiple phyla. Hence in the plant *Papaver somniferum* (opium poppy), morphine biosynthesis occurs through three different compartments (Beaudoin & Facchini, 2014). Briefly, companion cells produce most of the enzymes necessary for biosynthesis, which they transfer to the sieve elements, where dopamine is metabolized into different alkaloids. These alkaloids are finally transferred to the lactifers, which express the last three enzymes to produce morphine (Beaudoin & Facchini, 2014). Another example is vitamin D3, which can be acquired exogenously from food, or endogenously from the action of UVB on cholesterol derivatives in the skin. In order to produce functional 1,25‐vitamin D3, the vitamin D3 needs to undergo sequential modifications in liver hepatocytes and then in the kidney (Newmark *et al*, 2017). Finally, this process is also known for sex hormones. In mammals, females produce estrogen by converting cholesterol into androstenedione in theca cells. Androstenedione is transferred to granulosa cells, which express the aromatase that is necessary for the conversion into estrogen. This is known as the two‐cell, two‐gonadotropin hypothesis of estrogen production (Hillier *et al*, 1994). A similar process exists in embryonic, but not adult, testis, to produce testosterone. Indeed, fetal Leydig cells do not express the Hsd17b3 enzyme required to produce testosterone from androstenedione. This enzyme is only expressed by fetal Sertoli cells, hence the collaboration between different cell types (O'Shaughnessy *et al*, 2000; Shima *et al*, 2013). Therefore, this mechanism of cellular cooperation seems to be widely used in multicellular organisms. We had previously showed that HSPCs were closely associated with macrophages in the human FL (Cacialli *et al*, 2021). Here, we show that HSPCs are closely associated with both endothelial cells and macrophages in the E13 FL, confirming previously published results (Khan *et al*, 2016; Gao *et al*, 2022). Neutrophils were not found directly associated with these expansion pockets. As we saw previously, for vitamin D, the different cells involved in a metabolic pathway do not necessarily need to sit next to each other to perform biosynthesis. However, this first step of AA synthesis could also be performed by macrophages themselves as we show their expression of *Pla2g4c* to similar levels of *Pla2g4a* and *Pla2g4b* in neutrophils. While the deletion of neutrophils was not performed, Gao and colleagues deleted macrophages *in vivo* during embryonic development and observed a general decrease in hematopoiesis and a decrease in HSPC numbers (Gao *et al*, 2022). All these data contribute to highlight the role of myeloid cells in HSPC expansion during embryogenesis but was not enough to explain PGE2 synthesis.

We actually showed here that *Slco2b1* is important as an influx channel to import PGH2 into ECs, so that they can produce PGE2. Whereas all neutrophils and macrophages might express the necessary enzymes, we found that *slco2b1* expression was restricted to ECs in the CHT. Moreover, when *slco2b1*‐morphants showed a strong decrease in HSCs numbers, restoring *slco2b1* in ECs was the only way to rescue HSCs numbers, whereas overexpression in macrophages did not rescue their numbers. *abcc4*, the efflux transporter that releases PGE2 in the microenvironment, was also specifically expressed by ECs. Therefore, we can conclude that between 48 and 60hpf, CHT‐ECs are the unique source of PGE2 to expand HSCs, a role that is not surprising as CHT‐ECs also produce other cytokines controlling HSC expansion such as *kitlgb* (Mahony *et al*, 2016) and *osm* (Mahony *et al*, 2018).

During embryogenesis, many studies have shown the importance of primitive myeloid cells during the endothelium‐to‐hematopoietic transition. Indeed, macrophages seem to play different roles: aortic macrophages are responsible, together with neutrophils, to create a proinflammatory environment that promotes HSC specification, and the first round of expansion in the aortic niche (Espin‐Palazon *et al*, 2014). Macrophages are then responsible for the emigration of HSCs out of the subaortic environment, as they can remove the extracellular matrix to allow newly born HSCs to intravasate in the cardinal vein (Travnickova *et al*, 2015), an effect that could be mediated through metalloproteases (Theodore *et al*, 2017). Finally, a recent study revealed the essential role of VCAM‐1‐positive macrophages in HSC homing and retention in the CHT (Li *et al*, 2018). These macrophages and the ones responsible for PGH2 production likely come from the primitive, but not from the definitive wave, as recently demonstrated by fate‐mapping (Ferrero *et al*, 2021).

It has also been known for a long time, that macrophages, in mammals, contribute actively to adult hematopoiesis, either by helping red blood cells to enucleate (Rhodes *et al*, 2008) or to remove old erythrocytes in the spleen red pulp (Nagelkerke *et al*, 2018). Similar processes have been observed in the mouse FL and fetal spleen (Bertrand *et al*, 2006). In the bone marrow, α‐SMA‐positive macrophages interact tightly with HSCs. They express high levels of *Cox2* which function seems to be important to maintain the long‐term repopulation capability of HSCs, through the production of PGE2 (Ludin *et al*, 2012), a mechanism that seems similar to what we observe. However, in this study, the expression of *Ptges*, the enzyme that synthetizes PGE2 was not examined, and a contribution from the vascular niche cannot be excluded. Finally, it was recently shown that neutrophils are important for hematopoietic recovery after irradiation. By irradiating neutropenic animals, Ballesteros and colleagues could show that the recovery of most hematopoietic lineages was delayed (Ballesteros *et al*, 2020). Our zebrafish studies strongly point to the existence of a triad, composed of neutrophils, macrophages, and ECs that controls the fate of HSPCs. Our expression analysis of PGE2 biosynthetic components within mouse fetal liver cell subsets along with the evidence that macrophages, and neutrophils play a role in HSPC biology, suggests that such a collaborative mechanism for PGE2 production and regulation of HSPCs is likely conserved across vertebrates.

## Material and Methods

### Zebrafish husbandry

AB*zebrafish strains, along with transgenic strains and mutant strains, were kept in a 14/10 h light/dark cycle at 28°C. In this study we used the following transgenic lines: *Tg(mpeg1:GFP)* ^*gl22*^ (Ellett *et al*, 2011)*; Tg(Mmu.Runx1:NLS‐mCherry)* ^*cz2010*^ (mentioned as *runx1:mCherry*; Tamplin *et al*, 2015); *Tg(cmyb:GFP)* ^*zf169*^ (North *et al*, 2007)*; Tg(kdrl:Has.HRASmCherry)* ^*s896*^ (Chi *et al*, 2008)*; Tg(kdrl:GFP)* ^*s843*^ (Jin *et al*, 2005); *Tg(mpx:EGFP)* ^*i113*^ (Ellett *et al*, 2011); *Tg(kdrl:Gal4)* ^*bw9*^ *(*Kim *et al*, 2014 *); Tg(mpeg1:Gal4)* ^*gl24*^ (Ellett *et al*, 2011) *and Tg*(*cd45:CFP‐NTR*).

### Flow cytometry on zebrafish embryos

Transgenic zebrafish embryos were incubated with a Liberase‐Blendzyme3 (Roche) solution for 90 min at 33°C, then dissociated and resuspended in 0.9× PBS‐1% fetal calf serum, as previously described. We excluded dead cells by SYTOX‐red (Life Technologies) staining. Cell sorting was performed using an Aria II (BD Biosciences). Experiments were performed at the Cytometry Platform of the faculty of medicine.

### Quantitative real‐time PCR on zebrafish cells

Total RNA was extracted using RNeasy Mini Kit (Qiagen) and reverse transcribed into cDNA using a Superscript III kit (Invitrogen). Quantitative real‐time PCR (qPCR) was performed using KAPA SYBR FAST Universal qPCR Kit (KAPA BIOSYSTEMS) and run on a CFX connect real‐time system (Bio Rad). All Primers used are listed in Appendix Table S1.

### Generation of *cd45:CFP‐NTR* **transgenic animals**

The line was created using the Tol2 transposon system (Urasaki *et al*, 2006). Targeted cell ablation mediated by bacterial nitroreductase (NTR) was described previously (Curado *et al*, 2007). A DNA fragment containing CFP‐NTR was subcloned into a Tol2 vector that contained the zebrafish *cd45* promoter. The Tol2 construct and transposase mRNA were microinjected into 1‐cell stage embryos and founders were screened based on the presence of CFP in the progeny.

### Chemical treatments

The concentrations of all chemical treatments were chosen based on previous studies (North *et al*, 2007). All compounds used in these experiments were purchased from Sigma‐Aldrich. Zebrafish embryos were exposed for 12 h as a control in 0.2% dimethyl sulfoxide (DMSO), or 10 μm of PGE2 starting at 48 hpf. For metronidazole (MTZ) treatments, *cd45:CFP‐NTR* embryos were exposed to 10 mM MTZ in 0.2% DMSO, from 48 to 72 hpf. For treatment with PGE2‐related metabolites (AA, PGG2, PGH2, and PGE2) embryos were exposed to 10 μM.

### Identification of *slco2b1* mutant line

The *slco2b1* ^*sa37367*^ mutant line presents a point mutation T>C in the splice donor site at the end of exon4. The *slco2b1* ^*sa37367*^ line was purchased from the Zebrafish International Resource Center (ZIRC) (ZFIN ID: ZDB‐ALT‐160601‐5326). The *slco2b1* ^*sa37367*^ used in this paper was subsequently outcrossed with WT AB* for clearing of potential background derived from the random ENU mutagenesis from which this line was originated. Genotyping was performed by PCR of the *slco2b1* gene followed by sequencing. Genotyping primers are slco2b1‐F: ATACCAGACTCAACTCCAGC; slco2b1‐R: TGTCTATGTCGACATACAAGC.

### Morpholino injections

The *slco2b1*‐morpholino oligonucleotide (MOs) and control‐MO were purchased from GeneTools (Philomath, OR). MO efficiency was tested by reverse transcription polymerase chain reaction (RT–PCR) from total RNA extracted from ~10 embryos at 48 hpf. In all experiments, 12 ng of *slco2b1*‐MO were injected per embryo. Morpholinos and primer sequences are:

*Standard control‐MO* CCTCTTACCTCAGTTACAATTTATA;
*Slco2b1‐MO* CATCCATGCTTTTTATCCTTGCCTC;
*Slco2b1‐Forward* CTCCAGCTCTTCAGTCTCAG;
*Slco2b1‐Reverse* CTGTGTCTGGCAAAGGCT.

### Transient tissue‐specific overexpression of *slco2b1*

For the Tol2‐*UAS:slco2b1* construct, a Tol2 vector containing 4xUAS promoter, the *slco2b1* full cDNA, and a polyadenylation signal sequence were generated by subcloning. Next, to overexpress *slco2b1* in ECs and macrophages, we injected 25 pg of the Tol2‐*UAS:slco2b1* vector with 25 pg *tol2* transposase mRNA in *Tg(kdrl:Gal4)* or *Tg(mpeg1:Gal4)* embryos, respectively. The following primers were used to clone the full‐length *slco2b1* sequence: *Full‐Slco2b1‐Forward*: AAAGGATCCCTCCAGCTCTTCAGTCTCAG; *Full‐Slco2b1‐Reverse*: AAACTCGAGTGGCCTGTACAACTGCTTGC.

### Whole‐mount *in situ* hybridization and analysis

To generate digoxigenin and fluorescein‐labeled probes *cmyb*, *runx1*, *pu1*, *gata1*, *flk1*, and *rag1* were previously described (Cacialli *et al*, 2019; Cacialli *et al*, 2021; Mahony *et al*, 2021). Whole‐mount *in situ* hybridization (WISH) was performed on 4% paraformaldehyde‐fixed embryos. All injections were repeated three separate times. Analysis was performed using the unpaired Student *t*‐test or ANOVA multiple comparison test (GraphPad Prism). Embryos were imaged in 100% glycerol, using an Olympus MVX10 microscope. Oligonucleotide primers used to amplify and clone cDNA for the production of the *slco2b1* and *abcc4* ISH probe are *slco2b1*‐F:TGGTTGGGATTCCTGATAGC; *slco2b1*‐R:GGAAAGTGAAGCCACAAAGC; *abcc4*‐F:CCAGTCGACCTTCAGGA: *abcc4*‐R:CAGGAACAGGAAGCAAATCAAC.

### Confocal microscopy and immunofluorescence staining

Transgenic fluorescent embryos were embedded in 1% agarose in a glass‐bottom dish. Immunofluorescence double staining was performed with chicken‐anti‐GFP (1:400; Life Technologies) and rabbit‐anti‐phospho‐histone3 (pH3) antibodies (1:250; Abcam). We used AlexaFluor488‐conjugated anti‐chicken (1:1,000; Life Technologies) and AlexaFluor594‐conjugated anti‐rabbit (1:1,000; Life Technologies) secondary antibodies to reveal primary antibodies. Confocal imaging was performed using a Nikon inverted A1r spectral.

### Apoptotic‐cell detection

Apoptotic cells were detected by incubating live embryos in 10 μg/ml Acridine Orange (Cayman Chemical) in E3 for 30 min followed by 3 × 10 min‐washes in E3. Embryos were anesthetized with Tricaine and imaged.

### Time‐lapse imaging and analysis

For time‐lapse imaging, *Tg(cmyb:GFP*) embryos were anesthetized with 0.03% Tricaine (Sigma) and embedded in 1% agarose in a 60 mm glass‐bottom dish. Embryos were imaged at 28.5 °C with a confocal Nikon inverted A1r spectral. Scanning with 20× water immersion objective, z‐stacks were acquired with a step‐size of 7 μm within an interval of 10 min for 6 h in the control‐ and *slco2b1*‐morphants starting at ∼54hpf. The analysis of *cmyb:GFP* ^+^ cells in all experiments was performed using IMARIS image software.

### Enzyme‐Linked ImmunoSorbent assay (ELISA) for PGE2

We used the Human‐PGE2 ELISA Kit according to the manufacturer's instructions (Invitrogen). 50 DMSO or MTZ‐treated *cd45‐NTR:CFP* embryos were collected at 72 hpf. 50 embryos AB* injected by control and *slco2b1*‐morpholino were collected at 60 hpf. They were washed in ice‐cold PBS and homogenized in 500 μl TRIS buffer. Homogenate was spun down at 177 *g* for 4 min at 4°C to eliminate particles, and the supernatant was collected for ELISA. Assays were run in technical and biological triplicate.

### Mice

For experiments concerning cell sorting from FL, C57BL/6 mice were purchased from Charles River. Mice were cared for in accordance with Pasteur Institute guidelines in compliance with European animal welfare regulations; all animal studies were approved by Pasteur Institute Safety Committee according to the ethics charter approved by the French Agriculture ministry and to the European Parliament Directive 2010/63/EU. Experiments were performed under the license *02080.02*. For immunostaining experiments, FL was obtained from the Nef lab at our faculty of medicine (Geneva), as they study the development of gonads, under the license number *GE35*.

### Fetal liver cell preparation and sorting

E14.5 FLs were harvested, dissociated, and resuspended in Hanks' balanced‐salt solution (HBSS) supplemented with 1% FCS (Gibco). FL cells were depleted of Lineage^+^ cells by staining with biotinylated‐conjugated antibodies to lineage markers CD19 and Ter119 followed by incubation with streptavidin microbeads (Miltenyi Biotec). Depletion was performed on LS + MACS columns (Miltenyi Biotec), from which the negative fraction was recovered and stained for cell sorting. Dead cells were eliminated by Dead‐Live staining (Thermo Fischer) exclusion. FL subsets were purified by sorting with a FACS Aria III (Becton Dickinson). Cells were recovered in Eppendorf tubes for gene expression analyses. All antibodies were from BD Biosciences, eBioscience, Biolegend, Sony, Cell signaling Technologies, or R&D Systems. Antibodies either biotinylated or conjugated to fluorochromes (FITC, PE, PECy7, APC, APCCy7, BV510) were used against the following mouse antigens: Ly76 (TER‐119), Gr‐1 (L50‐823), CD19 (KMC8), cKit (2B8), Sca‐1 (D7), F4/80 (BM8), Gr‐1 (RB6‐8C5), CD45 (30F11), Flk1 (Avas12a1), CD31 (MEC13.3). Experiments were performed at the Cytometry facility of the Pasteur Institute.

### Quantitative real‐time PCR and analysis on mouse FL cell subsets

Cells were sorted in RLT Buffer (Qiagen) and frozen at −80°C. RNA was extracted using the RNeasy Micro Kit (Qiagen), and cDNA was obtained with PrimeScript™ RT Reagent Kit (Takara). A 7300 Real‐Time PCR System (Applied Biosystem) and Taqman technology (Applied Biosystem) were used for qRT–PCR. Statistical analysis was completed using one‐way ANOVA and multiple comparison tests. All primers used are listed in Appendix Table S2.

### Immunofluorescent stainings on fetal livers

Fetal livers were prepared in HOPE fixative (DCS Innovative) and embedded in low melting paraffin.

After deparaffination and rehydration, 2 μm sections were stained according to the following protocol. Between staining steps, slides were washed with Wash buffer (Dako, ref 53006). The following antibodies were used: rat‐anti‐mouse Major Basic Protein (homemade), rat‐anti‐F4/80 ‐FITC conjugated (BM8, Biolegend, ref 123108), rat‐anti‐mouse cKit‐ PE conjugate (2B8, Biolegend, ref 105807), and rat‐anti‐mouse CD31‐FITC conjugated (390, Biolegend, ref 102405). To perform the triple staining MBP‐cKit‐F4/80, sections were first incubated with goat‐anti‐mouse IgG Fab‐fragments (Jackson ImmunoResearch, 115‐007‐003) and goat serum to avoid subsequent unspecific binding. After washing, slides were incubated with MBP primary antibody and specific bounds visualized with a donkey‐anti rat AlexaFluor‐647 conjugated (Jackson ImmunoResearch,712‐605‐153). After wash, tissue sections were incubated with an avidin/biotin blocking system (Biolegend, ref 927301) and 10% normal rat serum to inactivate the endogenous biotin and block the unspecific bounds. Slides were incubated with F4/80‐FITC and cKit‐PE antibodies. To visualize specific staining, tissues were incubated with a rabbit‐anti‐FITC (Life, 711900) and a biotinylated mouse anti‐PE (Biolegend, 408103), followed by a goat‐anti‐rabbit AlexaFluor‐488 conjugated (Jackson ImmunoResearch, 111‐545‐144) and a Streptavidin‐Cy3 (Biolegend, ref 405215) incubation. To perform CD31‐cKit staining, tissue sections were incubated with an avidin/biotin blocking system (Biolegend, ref 927301) and 10% normal rat serum to inactivate the endogenous biotin and block the unspecific bounds. CD31‐FITC and cKit‐PE primary antibodies were applied to the sections. To visualize specific staining, the same secondary system was used as described above. Nuclei were stained with DAPI (Invitrogen, D1306). Slides were mounted with Fluoromount aqueous mounting medium (Sigma‐Aldrich, F4680). For each staining, we used three fetal livers from three different embryos and we acquired images from three different fields, the slides were scanned with the Panoramic 250 FLASH II (CaseViewer 3DHISTECH) Digital Slide Scanner at a 20× magnification.

## Author contributions

**Pietro Cacialli:** Conceptualization; data curation; formal analysis; methodology; writing – original draft; writing – review and editing. **Marie‐Pierre Mailhe:** Data curation. **Ingrid Wagner:** Data curation; methodology; writing – review and editing. **Doron Merkler:** Methodology; writing – review and editing. **Rachel Golub:** Data curation; formal analysis; funding acquisition; writing – original draft; writing – review and editing. **Julien Y Bertrand:** Conceptualization; supervision; funding acquisition; writing – original draft; writing – review and editing.

## Disclosure and competing interests statement

The authors declare that they have no conflict of interest.

## Supporting information




Appendix S1

Movie EV1

Movie EV2

Source Data for Expanded View and Appendix

Source Data for Figure 1

Source Data for Figure 2

Source Data for Figure 3

Source Data for Figure 4

Source Data for Figure 5

Source Data for Figure 6

Source Data for Figure 7

## Data Availability

All raw data are freely accessible on the following link: https://doi.org/10.26037/yareta:caj5wsstqzaybovbnierjy4lqa.
