# Supplementary material for: Synergistic prostaglandin E synthesis by myeloid and endothelial cells promotes fetal hematopoietic stem cell expansion in vertebrates
Source: EMBO J. 2022 Aug 4;41(19):e108536. doi: 10.15252/embj.2021108536 (PMC9531293; doi:10.15252/embj.2021108536)
Supplement: Supplementary file 1 — Appendix S1 [file EMBJ-41-e108536-s010.pdf]

Appendix

Synergistic prostaglandin E synthesis by myeloid and endothelial cells promotes fetal hematopoietic stem cell expansion in vertebrates

Pietro Cacialli[1], Marie-Pierre Mailhe[2], Ingrid Wagner[1], Doron Merkler[1,3], Rachel Golub[2,4] and Julien Y. Bertrand[1,5]

Table of content

Appendix Figure S1. The prostaglandin transporters *slco2b1* and *abcc4* are expressed in the CHT niche.

Appendix Figure S2. A new zebrafish model for myeloid ablation.

Appendix Figure S3. The *cd45* 7.6kb promoter is not active in HSPCs.

Appendix Figure S4. Metronidazole (MTZ) treatment depletes macrophages and neutrophils in *cd45:CFP-NTR* embryos.

Appendix Figure S5. Myeloid ablation decreases PGE2 levels in the CHT.

Appendix Figure S6. qPCR screening of genes involved in the PGE2 synthesis pathway after myeloid ablation.

.

Appendix Figure S7. MTZ treatment on AB* embryo does not affect HSCs.

Appendix Figure S8. The loss of HSCs after myeloid ablation cannot be rescued by AA or PGG2 treatments.

Appendix Figure S9. *Slco2b1*$^{-/-}$ mutants present a decrease of HSPCs in the CHT.

Appendix Figure S10. *Slco2b1*-deficiency does not affect primitive erythropoiesis, vasculogenesis and myelopoiesis

Appendix Figure S11 Morpholino-mediated knockdown of *slco2b1* expression fully phenocopies *slco2b1*$^{-/-}$ mutants.

Appendix Figure S12. *Slco2b1*-deficiency does not induce apoptosis of HSCs.

Appendix Figure S13. The loss of HSPCs in *slco2b1*-deficient embryos can only be rescued by PGE2 treatment.

Appendix Figure S14. *Slco2b1*-overexpression in ECs or macrophages through the Gal4:UAS system.

Appendix Figure S15. *Slco2b1*-overexpression in macrophages cannot rescue the loss of HSPCs in *slco2b1*-morphants.

Appendix Figure S16. The PGE2 synthesis pathway is conserved in the mouse fetal liver.

Appendix Table S1. Primer used for quantitative real time PCR of zebrafish expressed genes.

Appendix Table S2. Primer used for quantitative real time PCR of mouse expressed genes.

Appendix Figure S1. The prostaglandin transporters *slco2b1* and *abcc4* are expressed in the CHT niche.

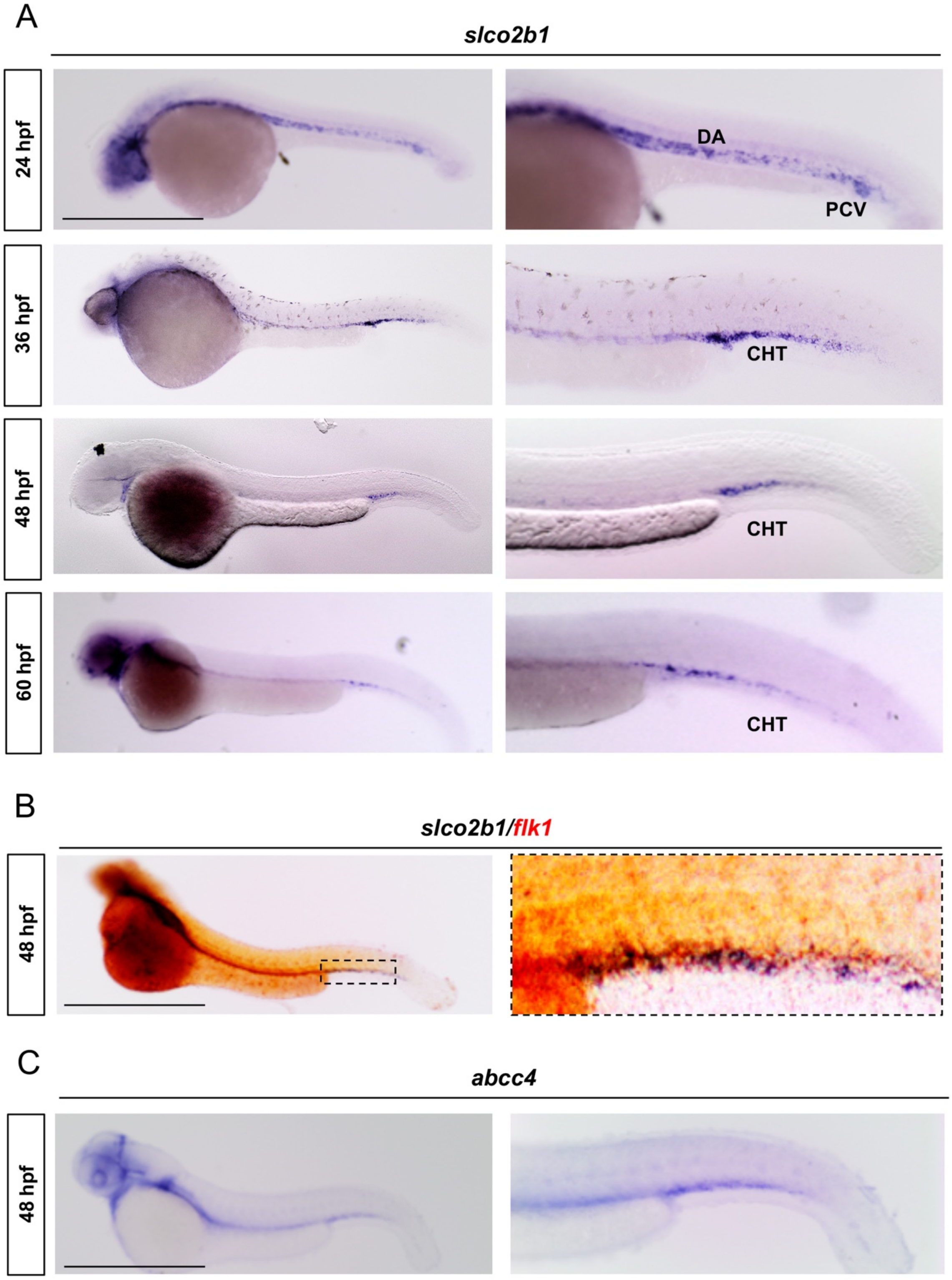

(A) WISH for *slco2b1* expression during zebrafish embryo development at different stages. (B) Double WISH for *flk1*(red) and *slco2b1* (black) at 48hpf. (C) *abcc4* expression at 48hpf. Scale bar 500µm (A-B-C).

Appendix Figure S2. A new zebrafish model for myeloid ablation.

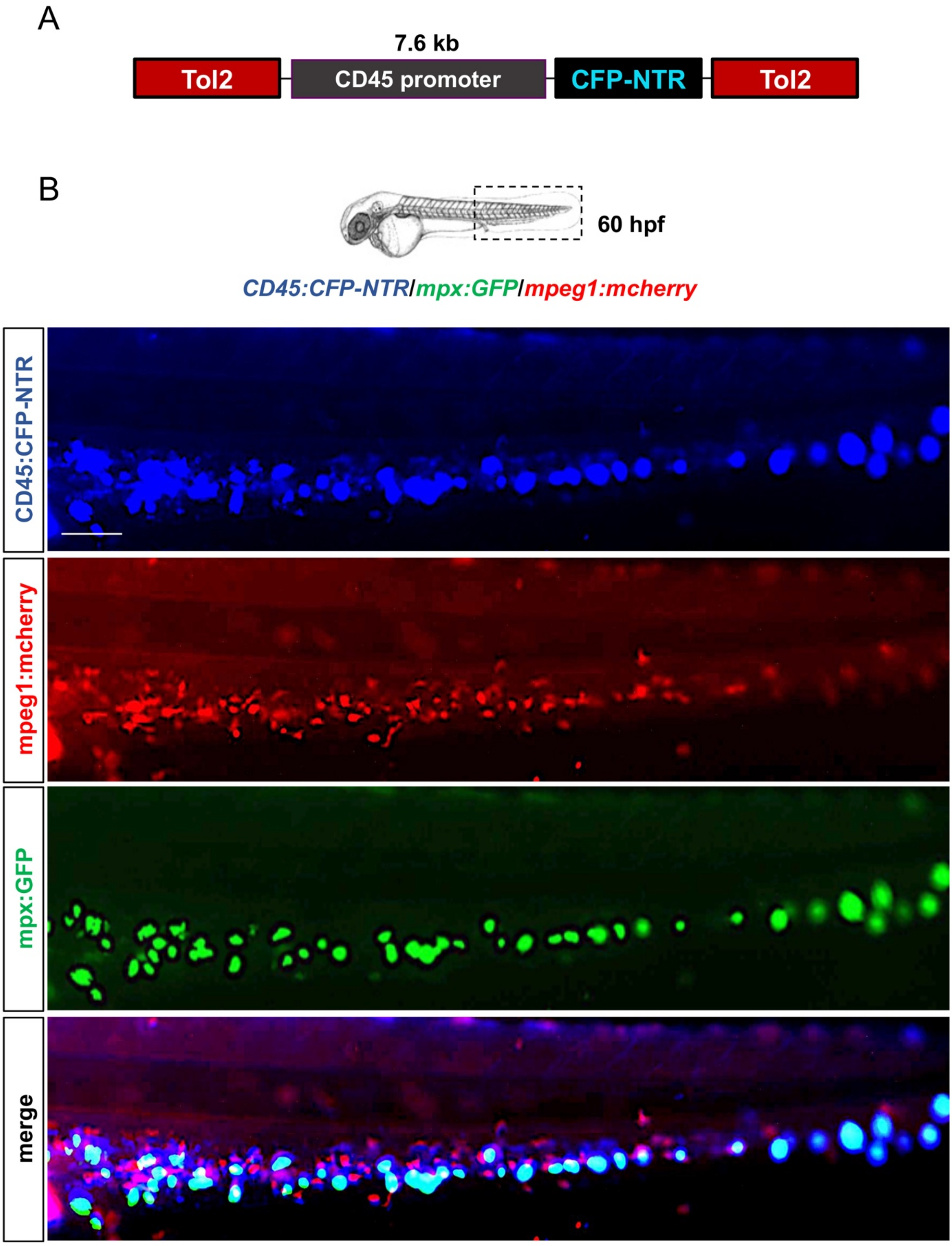

(A) the *cd45:CFP-NTR* transgenic construct. (B) Fluorescence imaging of the CHT in a triple transgenic *cd45:CFP-NTR;mpeg1:mCherry;mpx:eGFP* embryo at 60hpf. All blue (CFP^+) cells are either *mpeg1:mCherry*$^+$ or *mpx:eGFP*$^+$.

Appendix Figure S3. The *cd45* 7.6kb promoter is not active in HSPCs.

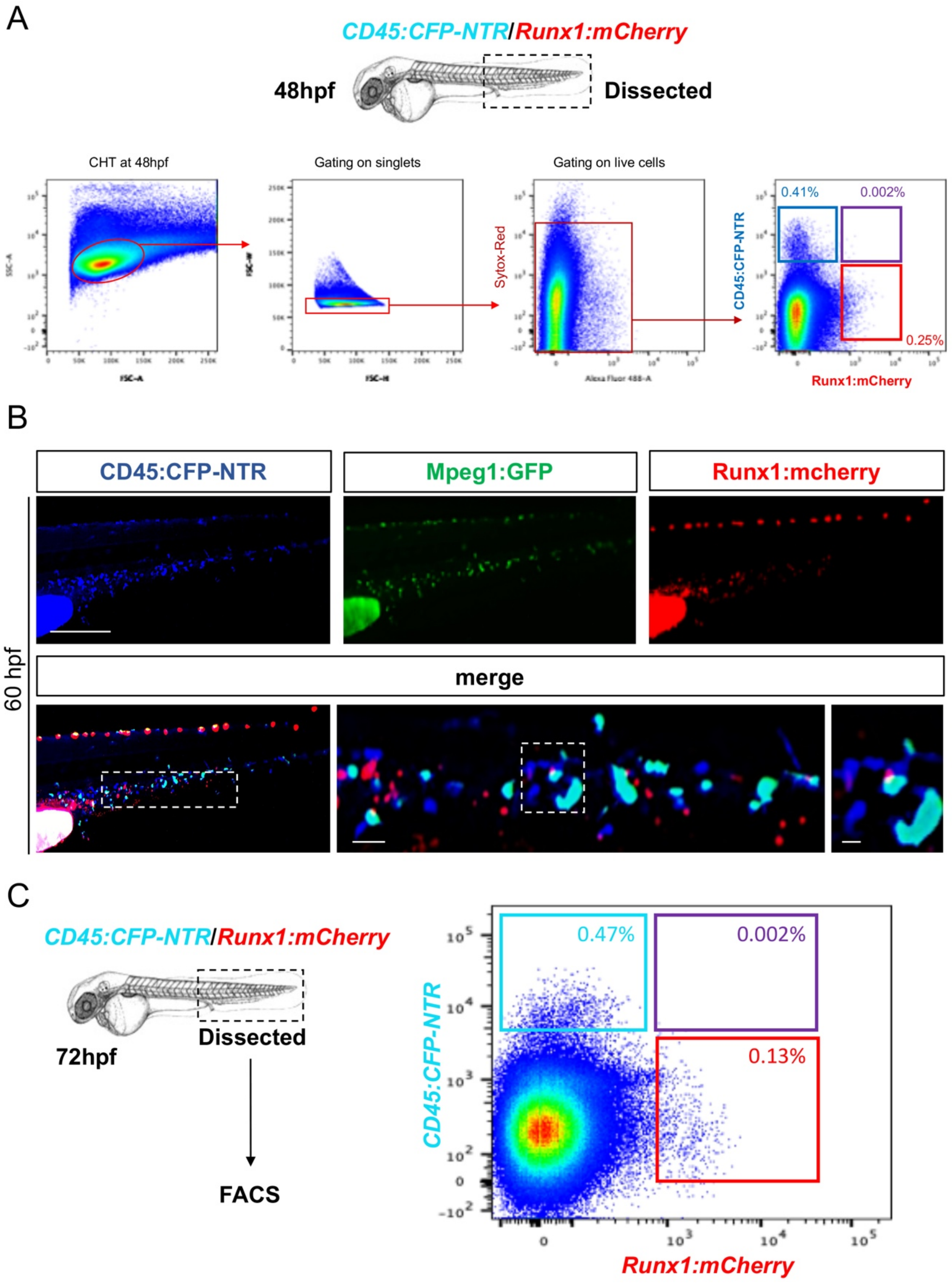

(A) Cytometry performed on double transgenic *cd45:CFP-NTR;runx1:mCherry* embryos at 48hpf. (B) Fluorescence live-imaging of the CHT of triple transgenic *cd45:CFP-NTR;runx1:mCherry;mpeg1:eGFP* embryos at 60hpf. (C) Cytometry performed on double transgenic embryos *cd45:CFP-NTR/runx1:mCherry* at 72hpf. The gating strategy was identical to (A).

Appendix Figure S4. Metronidazole (MTZ) treatment depletes macrophages and neutrophils in *cd45:CFP-NTR* embryos.

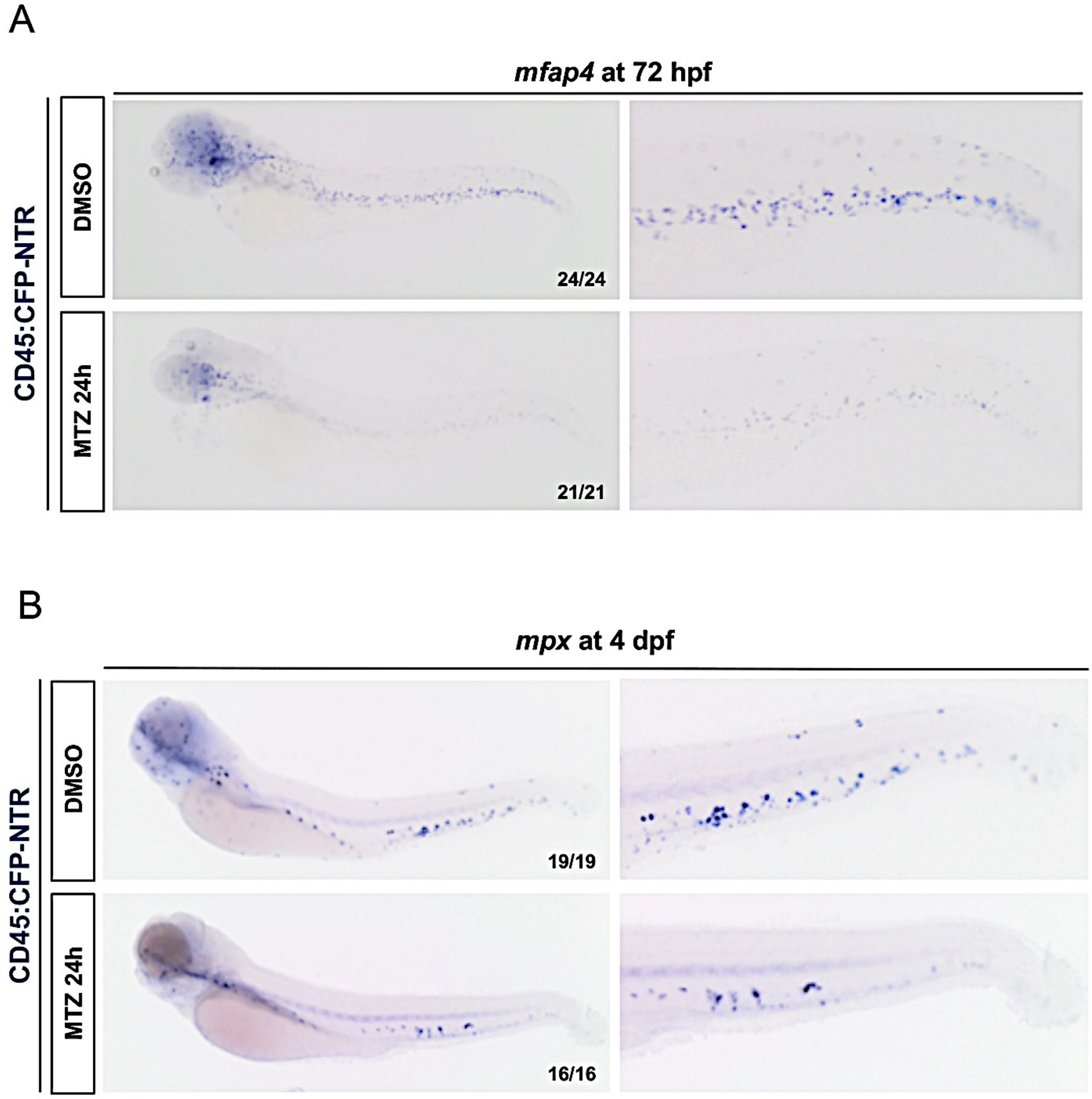

(A) WISH for *mfap4* expression in *cd45:CFP-NTR* embryos at 72hpf treated with DMSO and/or MTZ for 24h. (B) WISH for *mpx* expression in *cd45:CFP-NTR* embryos at 4dpf treated with DMSO and/or MTZ for 24h.

Appendix Figure S5. Myeloid ablation decreases PGE2 levels in the CHT.

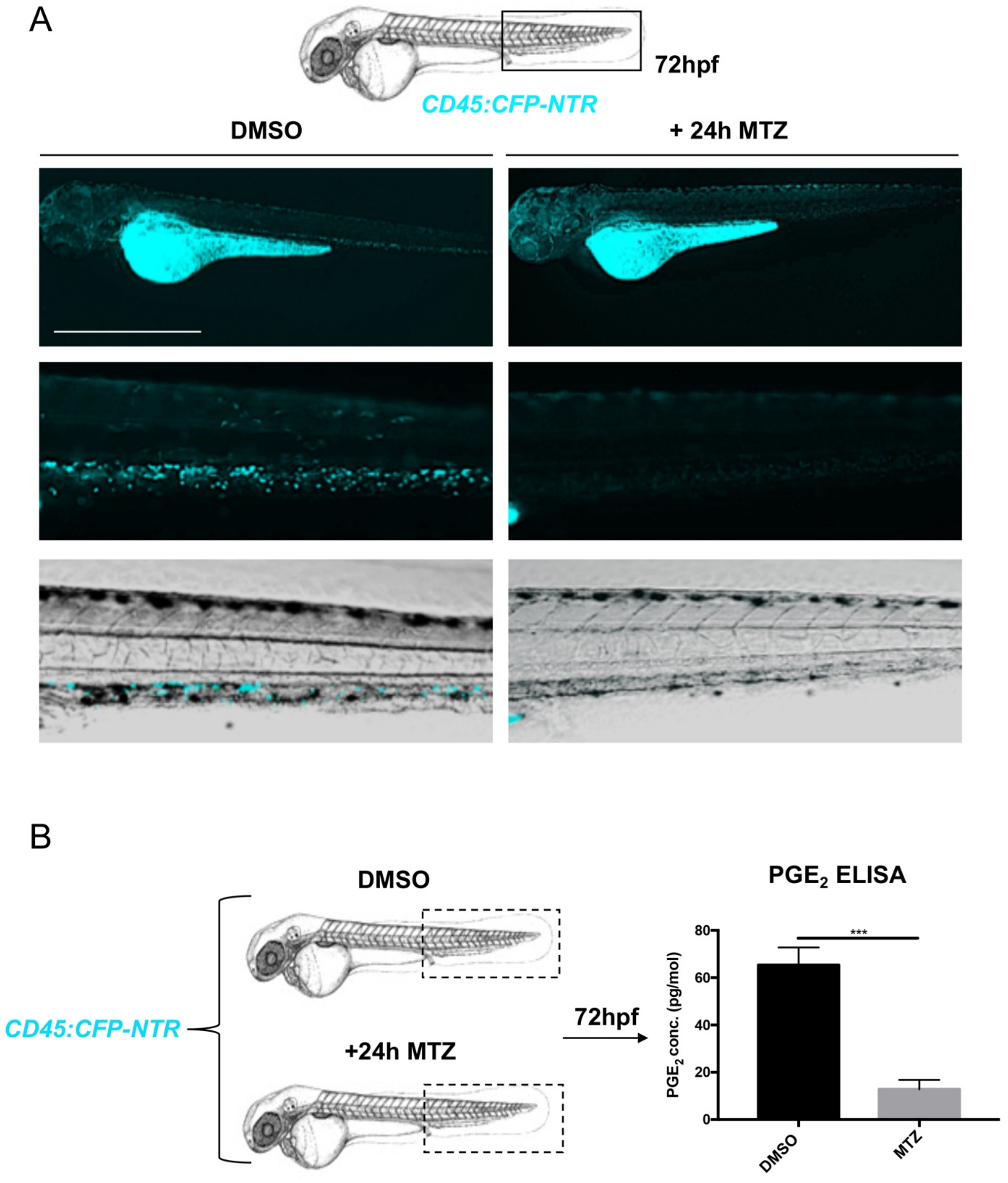

(A) Fluorescence imaging of a *cd45:CFP-NTR* embryo treated DMSO and/or MTZ for 24 hours. (B) Experimental outline to measure PGE2 levels by ELISA-kit in 72hpf *cd45:CFP-NTR* embryos after 24 hours of DMSO or MTZ treatments. Quantification of PGE2. The statistical analysis was completed using an unpaired two tailed t test ***P < .001. Scale bar 500µm (A)

Appendix Figure S6. qPCR screening of genes involved in the PGE2 synthesis pathway after myeloid ablation.

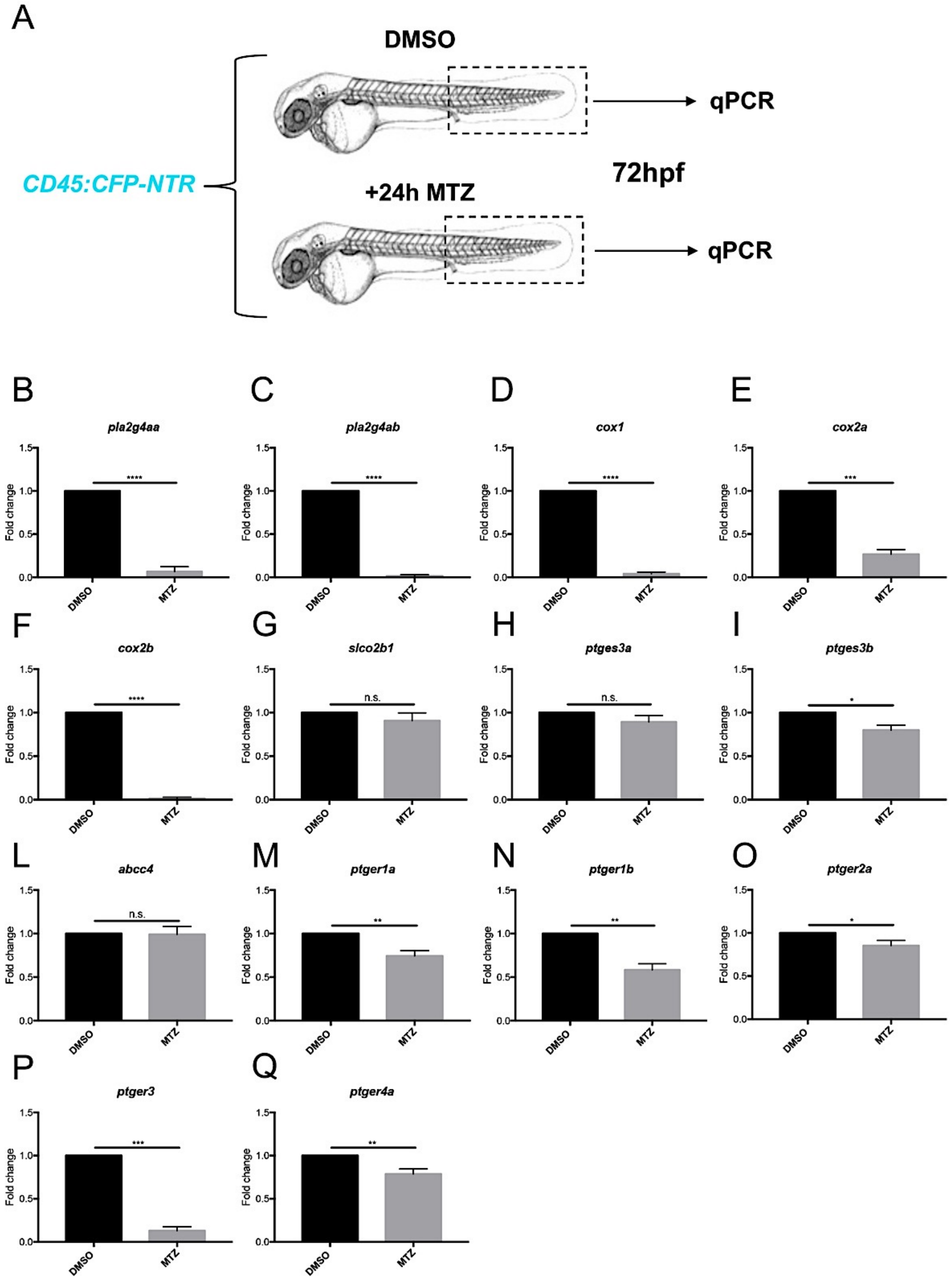

(A) Experimental outline of qPCR analysis after myeloid ablation in *cd45:CFP-NTR* embryos by MTZ treatment. (B-C) The expression of phospholipases *pla2g4aa* and *pla2g4ab* was severely reduced after myeloid ablation. (D-E-F) So was the expression of cyclooxygenases cox1, cox2a and cox2b. (G-H-I-L) No difference of expression for the prostaglandin transporters *slco2b1* and *abcc4*, nor for the prostaglandin synthase *ptges3a*. *ptges3b* was midly reduced after myeloid ablation. (M-N-O-P-Q) Significative reduction for the different PGE2 receptors after myeloid ablation.

Appendix Figure S7. MTZ treatment on Ab* embryo does not affect HSPCs.

A

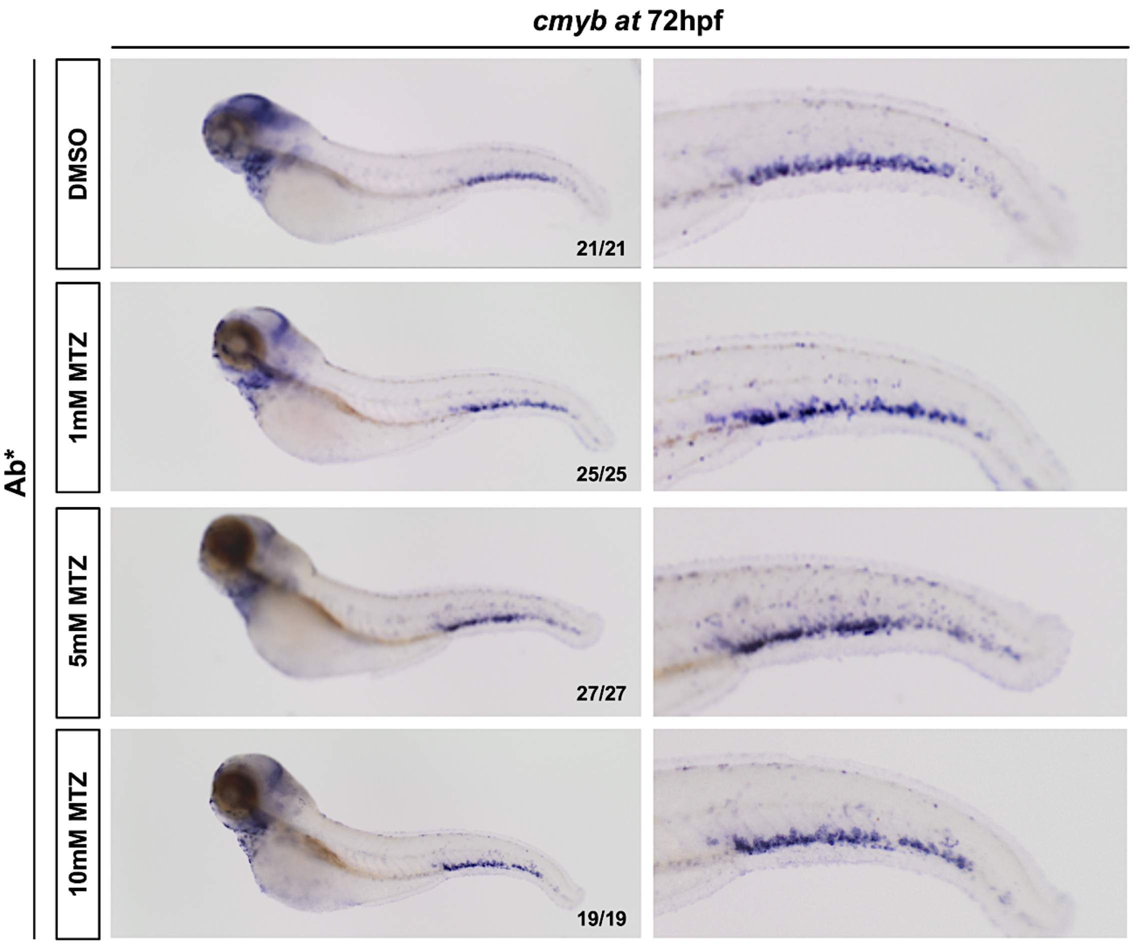

(A) WISH for *cmyb* in AB* embryos at 72hpf after 24h of DMSO and/or MTZ treatment using different doses.

Appendix Figure S8. The loss of HSPCs after myeloid ablation cannot be rescued by AA or PGG2 treatments.

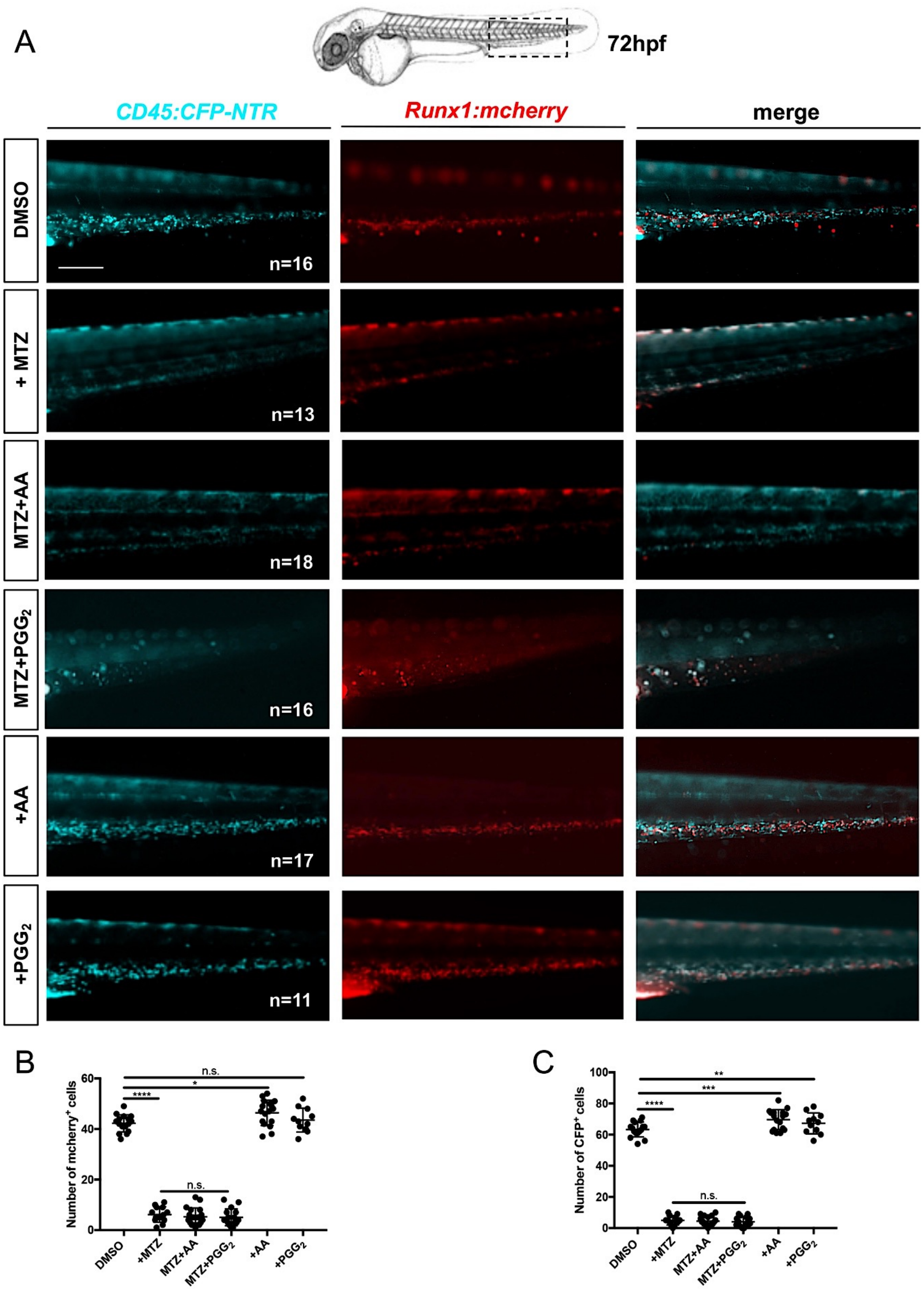

(A) Fluorescence imaging in the CHT of double transgenic *cd45:CFP-NTR;runx1:mcherry embryos* in DMSO and after treatment with MTZ and AA or PGG2. (B) Quantification of *runx1:mcherry* positive-cells in double transgenic *cd45:CFP-NTR;runx1:mcherry* embryos in DMSO and after treatment with MTZ and AA or PGG2. (C) Quantification of *cd45:CFP positive* cells in double transgenic *cd45:CFP-NTR;runx1:mcherry* embryos in DMSO and after treatment with MTZ and AA or PGG2. Statistical analysis was completed using one-way ANOVA, multiple comparison test. **P<.01; ****P<.0001.

Appendix Figure S9. *Slco2b1$^{-/-}$* mutants present a decrease of HSPCs in the CHT.

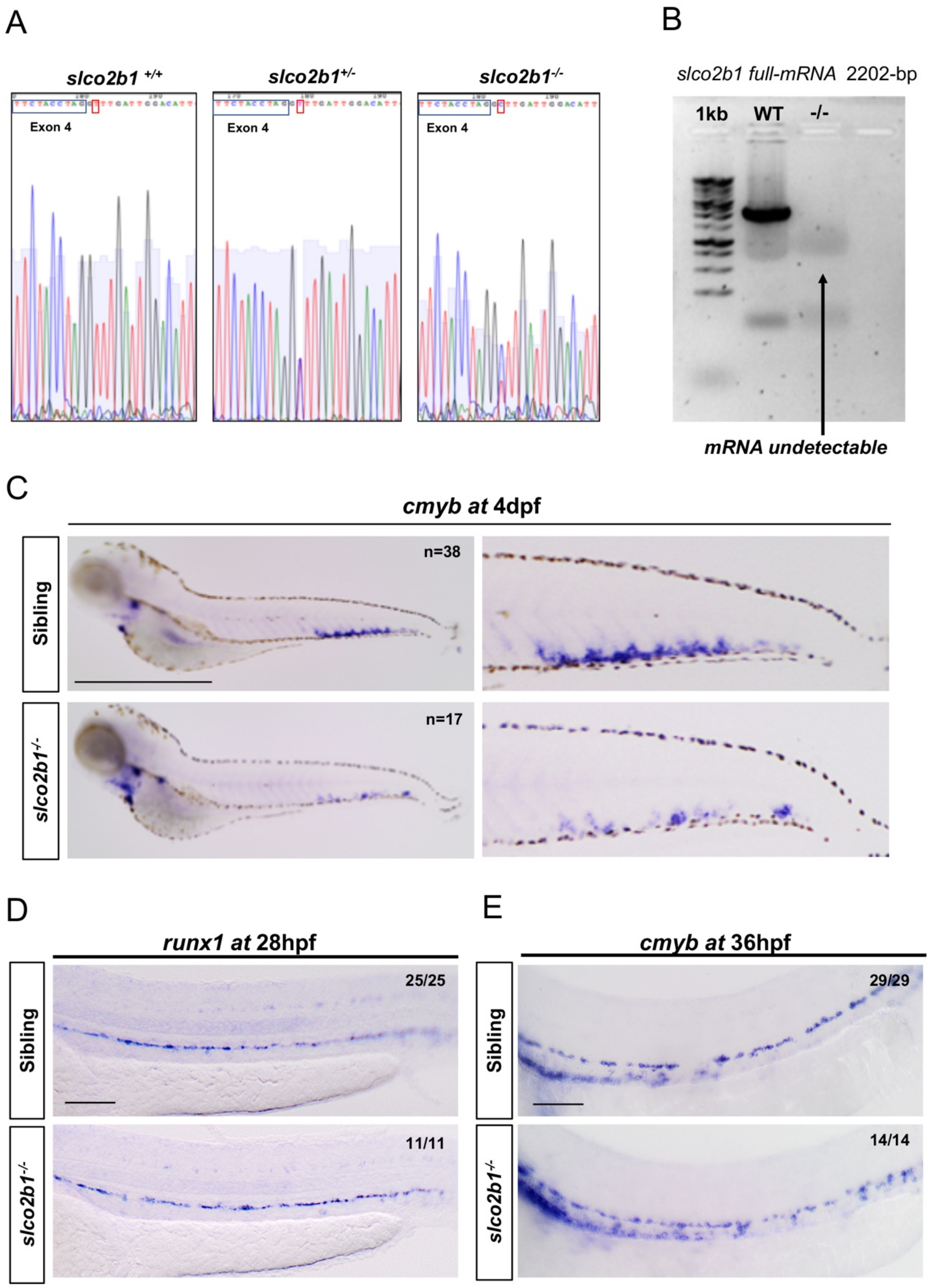

(A) Sequencing of PCR products to genotype the *slco2b1^{sa37367}* mutant line. This mutant presents a point mutation T>C in the splice donor site at the end of exon 4. (B) Detection of *slco2b1* full-mRNA in wild type and *slco2b1$^{-/-}$*. (C) WISH for cmyb expression at 4dpf in siblings and *slco2b1$^{-/-}$* embryos. (D) WISH for runx1 expression at 28hpf in siblings and *slco2b1$^{-/-}$* embryos. (E) WISH for cmyb expression at 36hpf in siblings and *slco2b1$^{-/-}$* embryos. Scale bar 500µm (C); 200µm (D-E).

Appendix Figure S10. The *slco2b1*-deficiency does not affect primitive erythropoiesis, vasculogenesis or myelopoiesis.

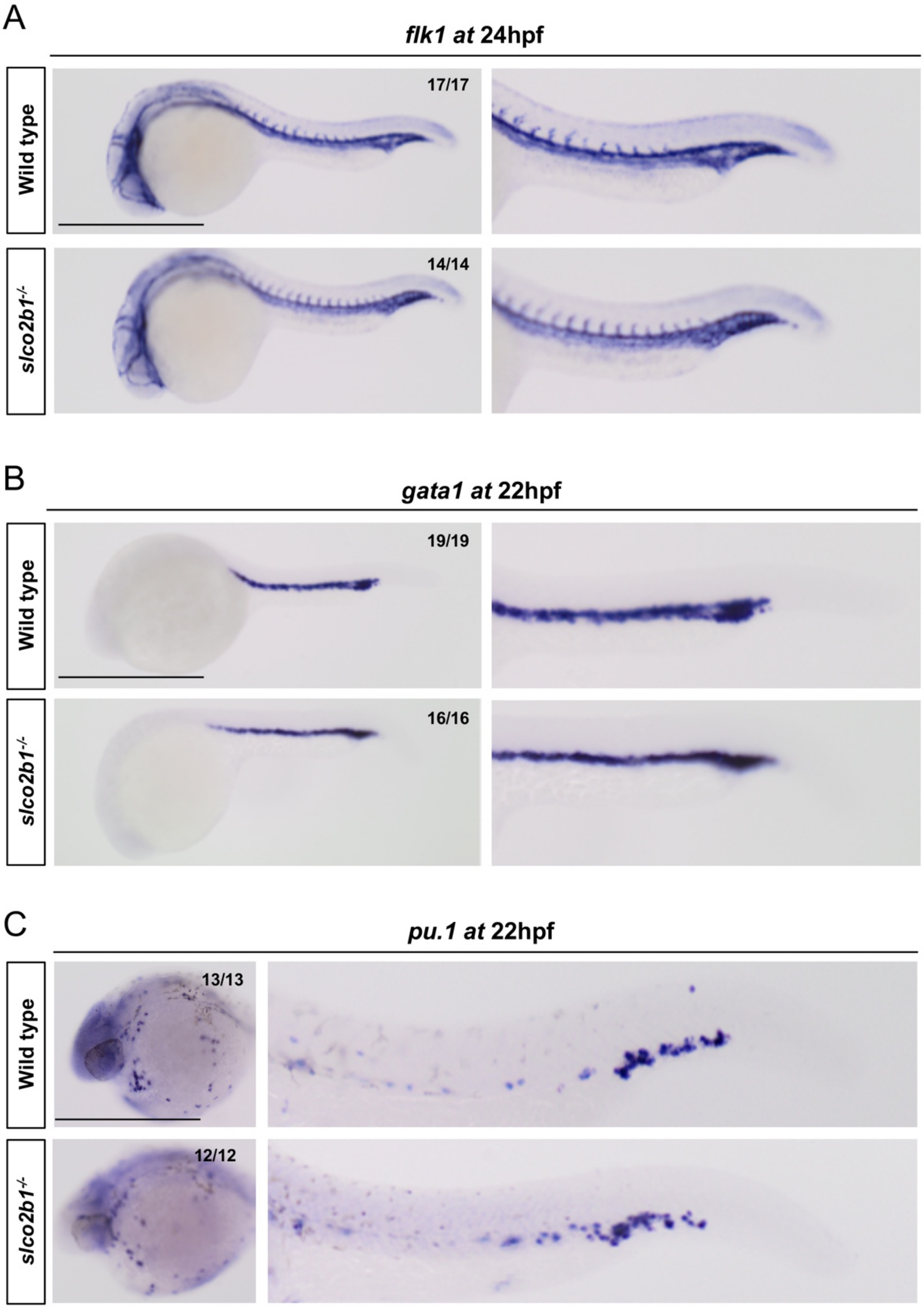

(A) WISH for *kdrl* at 24hpf in siblings and *slco2b1*$^{-/-}$ embryos. (B) WISH for *gata1* at 22hpf in siblings and *slco2b1*$^{-/-}$ embryos. (C) WISH for pu.1 at 22hpf in siblings and *slco2b1*$^{-/-}$ embryos. Scale bar 500µm (a-b-c).

Appendix Figure S11. Morpholino-mediated knockdown of *slco2b1* expression fully phenocopies *slco2b1*$^{-/-}$ mutants.

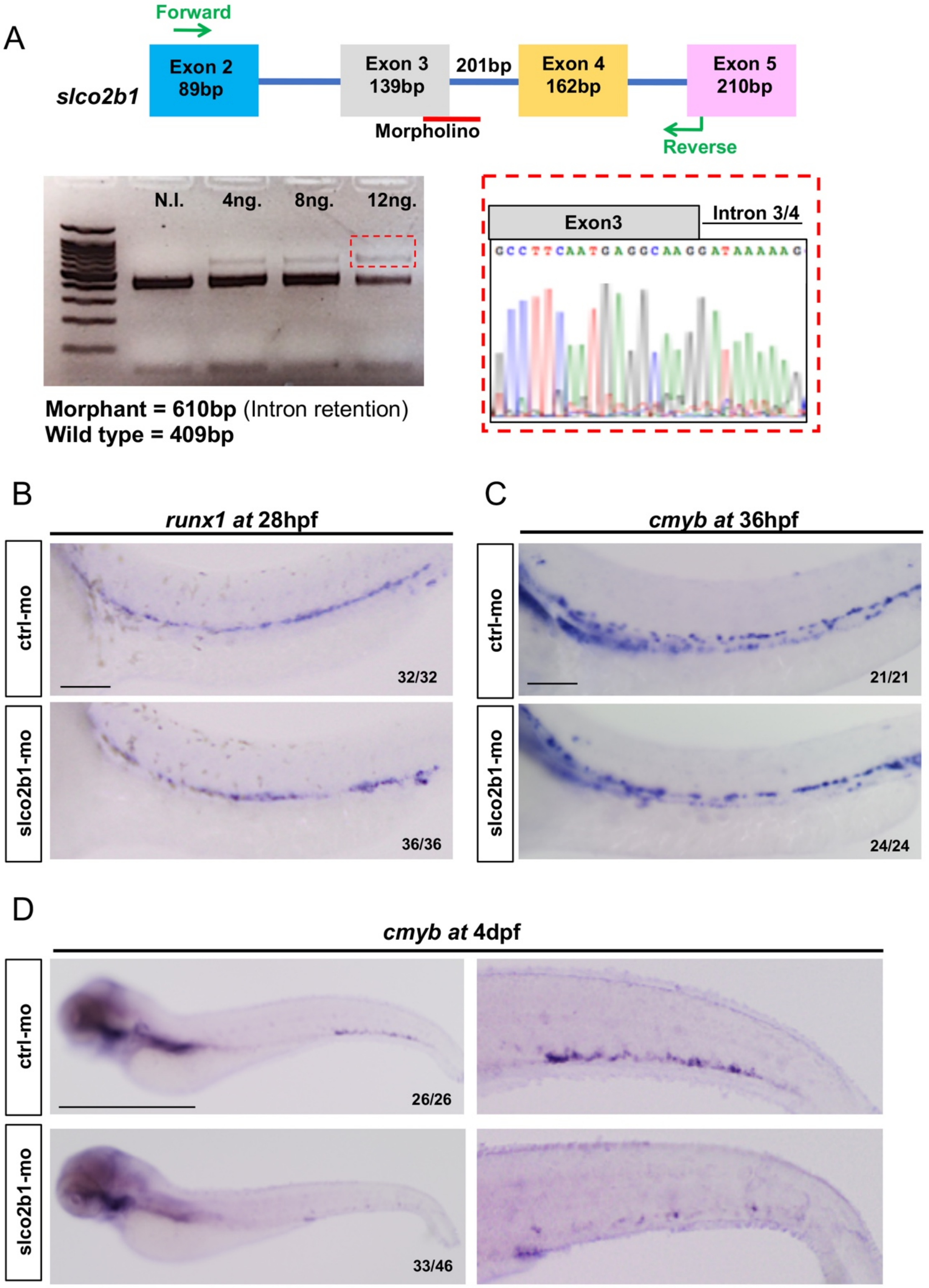

(A) Schematic of MO-retention targeting intron/exon junctions in *slco2b1*. Validation of the *slco2b1*-MO that induces intron-3 retention. The RT-PCR was performed on mRNA/cDNA obtained at 48hpf from pools of 10 embryos. The PCR products were sequenced using the forward primer. (B) Wish

for *runx1* expression at 28hpf in control and *slco2b1*-morphants. (C) WISH for cmyb expression at 36hpf control and *slco2b1*-morphants. (D) WISH for cmyb expression at 4dpf control and *slco2b1*-morphants. Scale bar 200µm (b-c); 500µm (d).

Appendix Figure S12 The *slco2b1*-deficiency does not induce apoptosis of HSPCs.

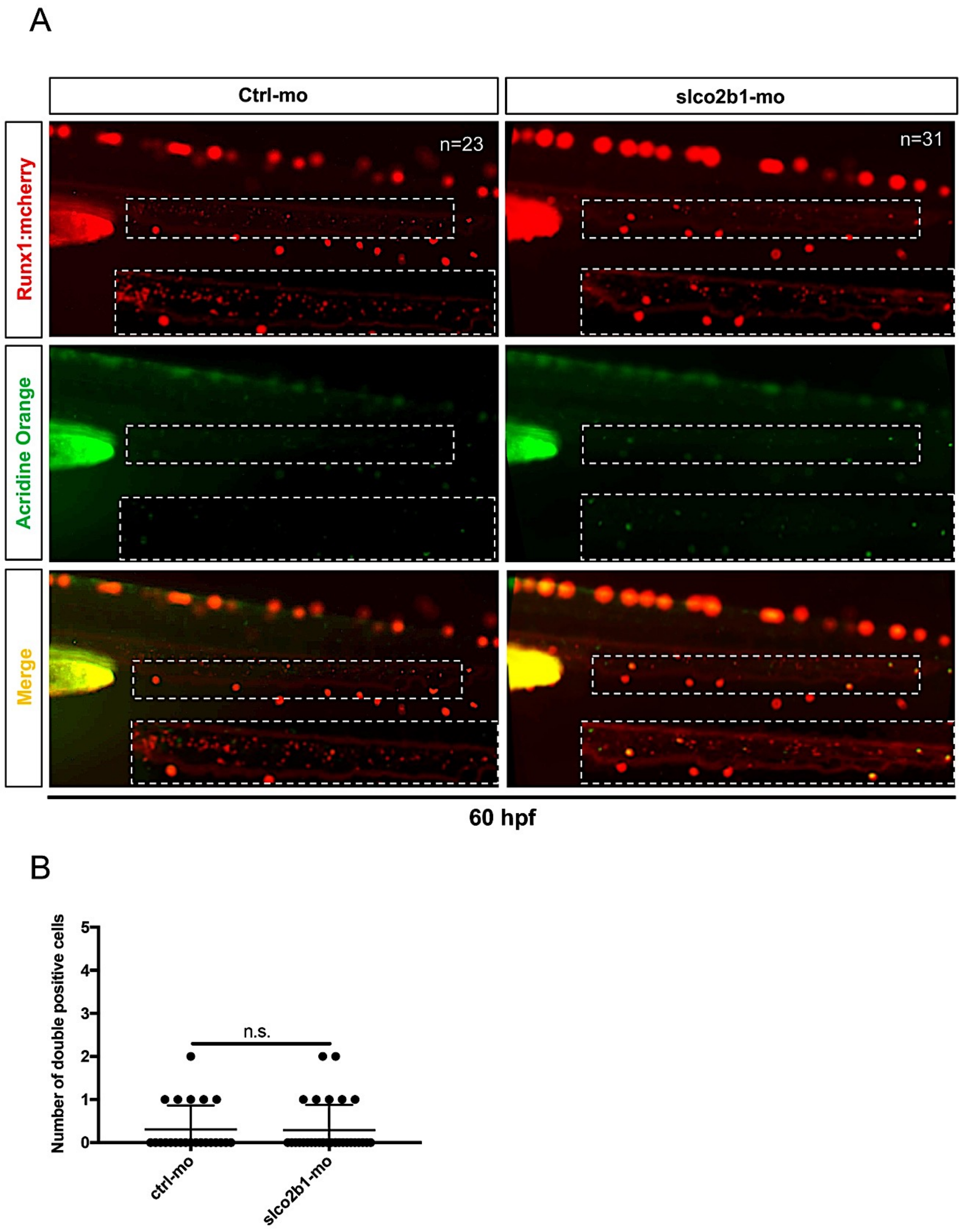

(A) Acridine orange stainings of *runx1:mCherry* embryos injected with control and *slco2b1*-morpholinos. (B) Quantification of the number of double-positive cells. Statistical analysis was completed using unpaired t-test.

Appendix Figure S13. The loss of HSPCs in *slco2b1*-deficient embryos can only be rescued by PGE2 treatment.

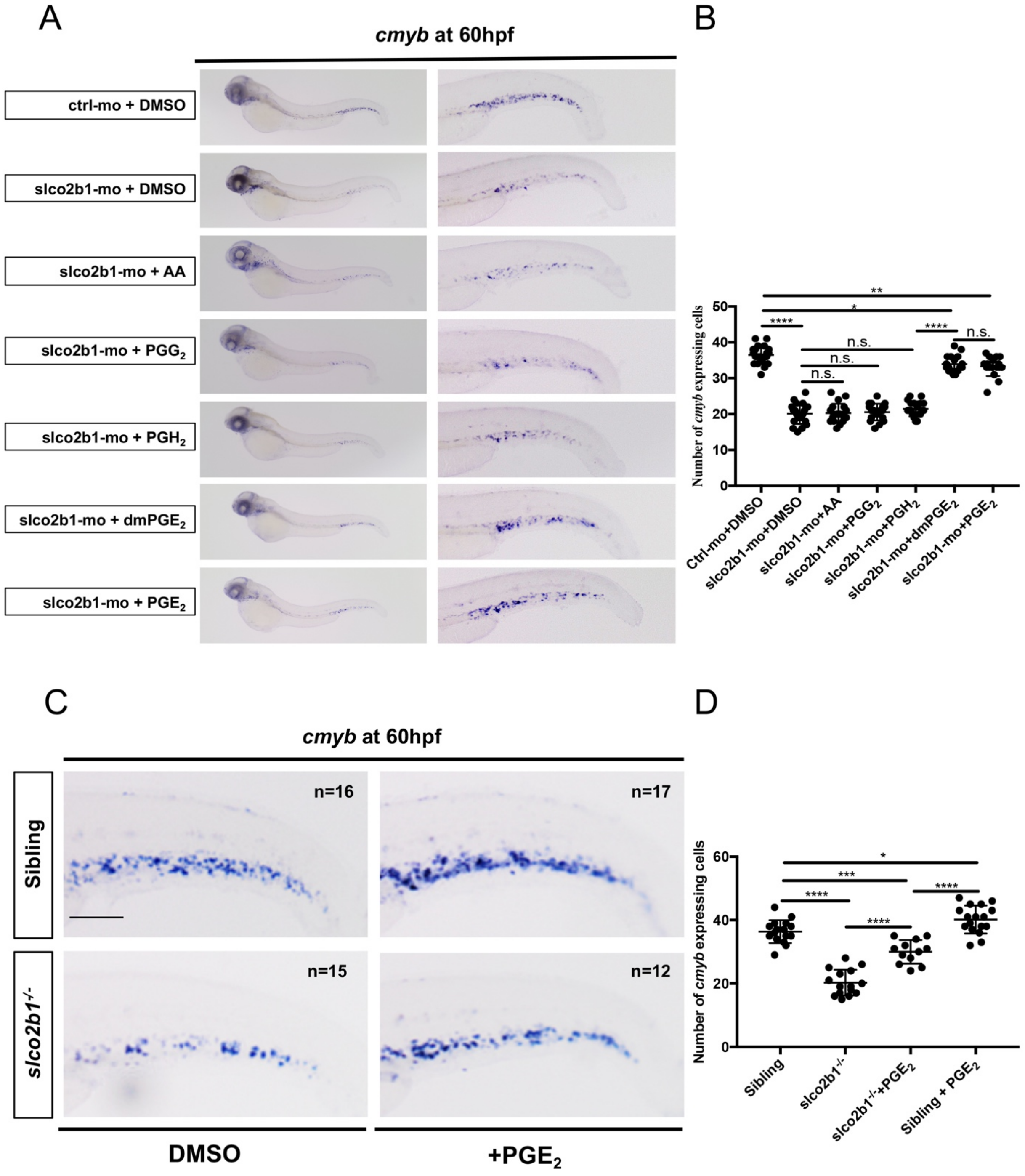

(A) WISH for cmyb expression at 60hpf in embryos injected with control- or slco2b1- morpholinos, and treated with DMSO and/or AA, PGG2, PGH2, PGE2. (B) Quantification of the number of *cmyb*-expressing cells. (C) WISH for *cmyb* in Sibling and slco2b1$^{-/-}$ treated with DMSO and or $PGE_{2.}$ (D) Quantification of the number of *cmyb*-expressing cells. Statistical analysis was completed using one-way ANOVA, multiple comparison test. *P< .01; ***P < .001; ****P < .0001. Scale bar 200μm (A-C).

Appendix Figure S14. Tissue-specific *slco2b1*-overexpression in ECs or macrophages through the Gal4:UAS system.

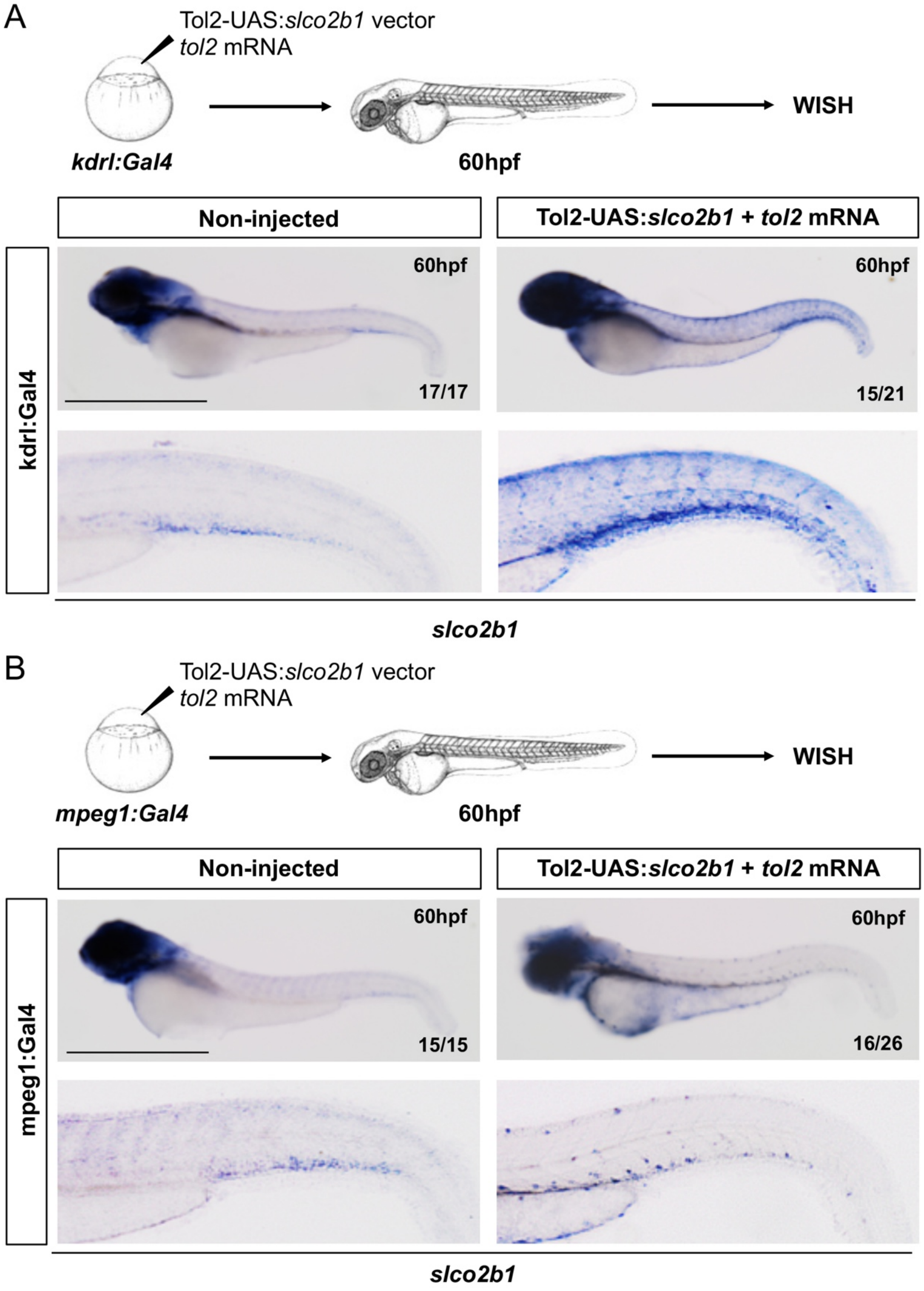

(A) WISH for *slco2b1* at 60hpf in *kdrl:Gal4*$^{+}$ embryos non-injected or after co-injection with the *Tol2-UAS:slco2b1* vector and *tol2* mRNA. (B) WISH for *slco2b1* at 60hpf in *mpeg1:Gal4*$^{+}$ embryos non-injected and after co-injection with the *Tol2-UAS:slco2b1* vector and *tol2* mRNA.

Appendix Figure S15. The *slco2b1*-overexpression in macrophages cannot rescue the loss of HSPCs in *slco2b1*-morphants.

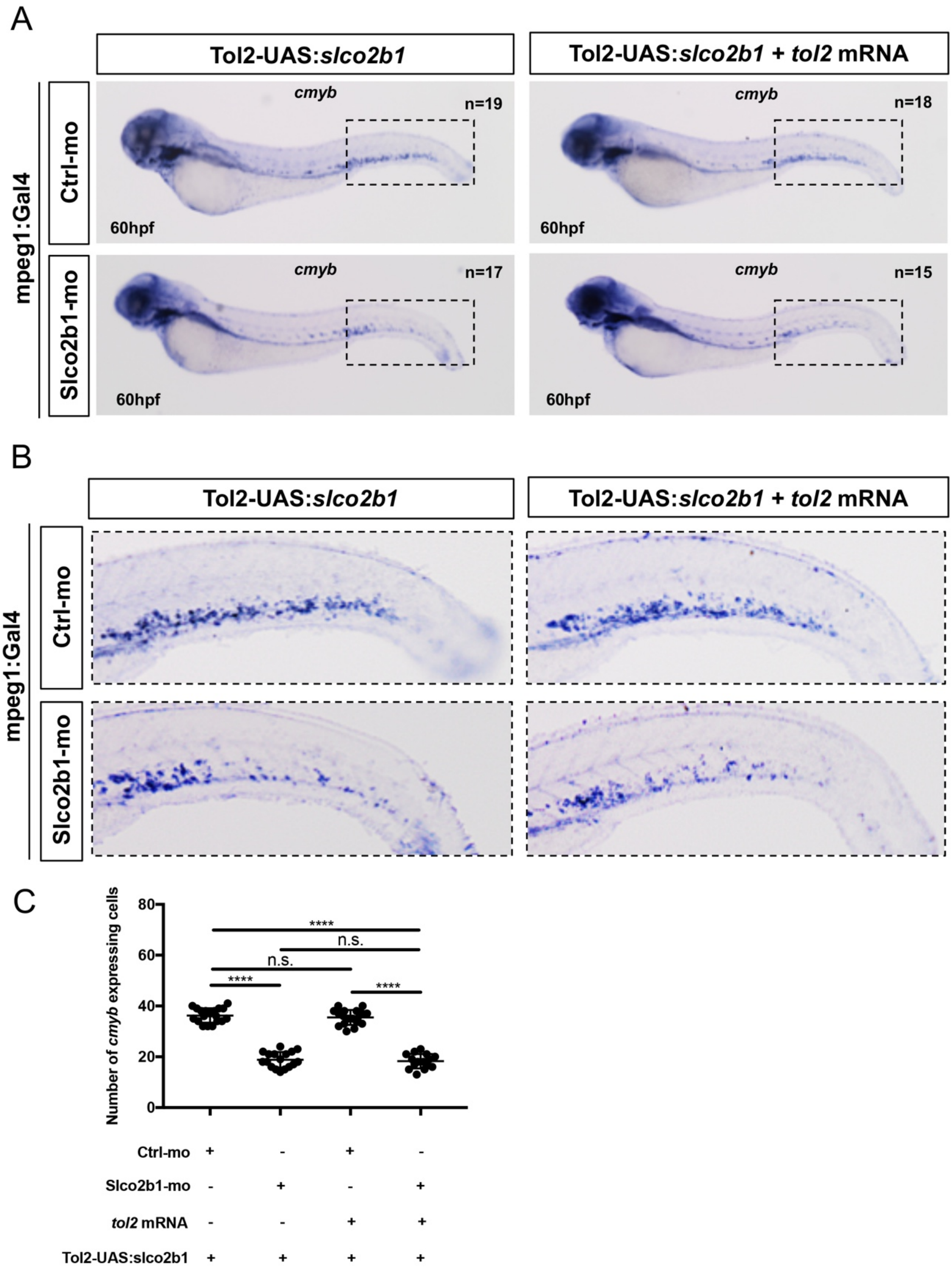

(A) WISH for cmyb at 60hpf in *mpeg1:Gal4*$^{+}$ embryos injected with control and *slco2b1*-morpholinos and/or co-injected with the *Tol2-UAS:slco2b1* vector and *tol2* mRNA. (B) magnification of CHT regions. (C) Quantification and statistical analysis were completed using one-way ANOVA, multiple comparison test. ****P<.0001.

Appendix Figure S16. The PGE2 synthesis pathway is conserved in the mouse fetal liver.

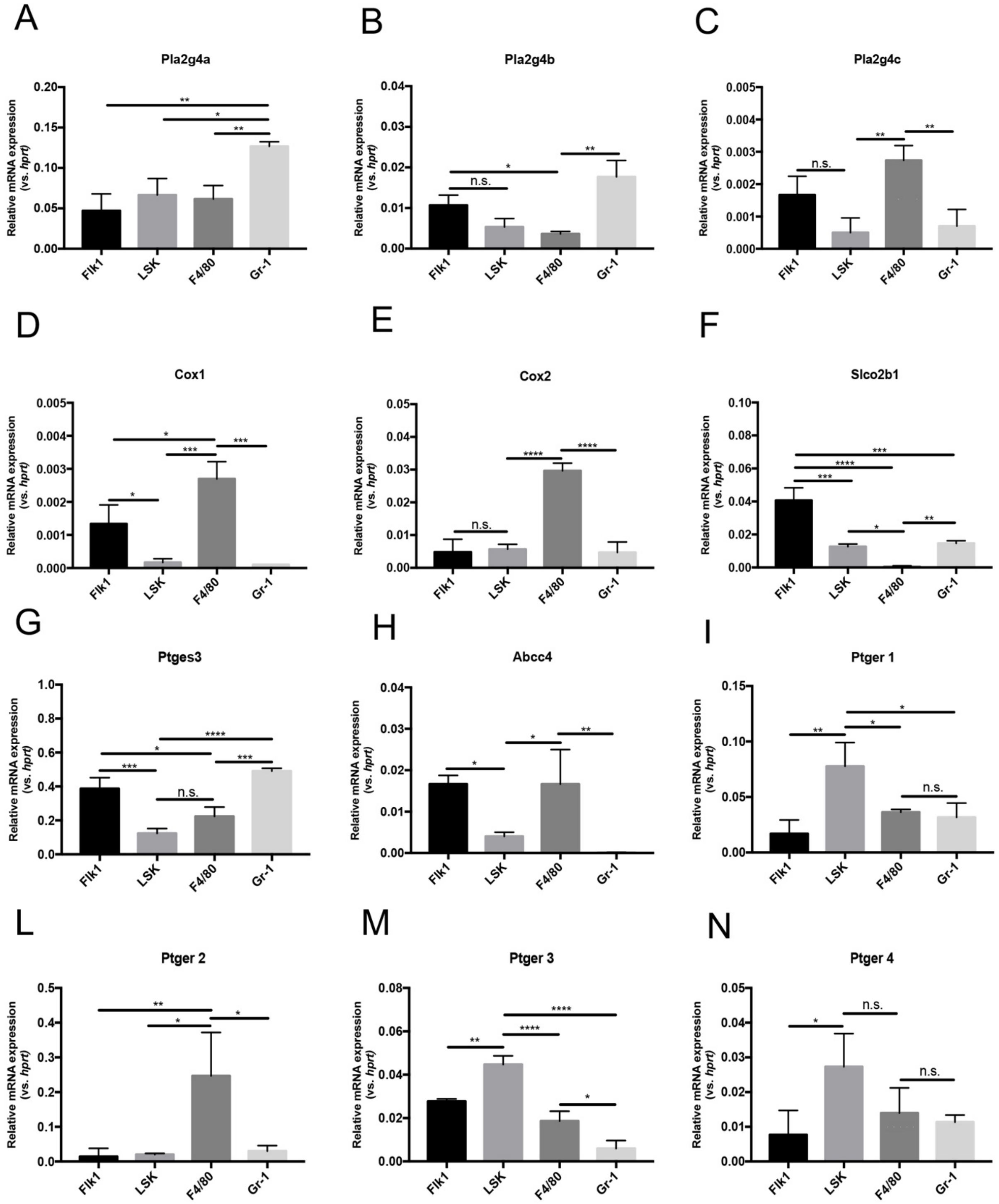

qPCR analysis on FACS-sorted cells from mouse fetal livers at Embryonic day (E) 13.5 by using various combinations of antibodies. HSCs were isolated based on their Lin-Sca1+cKit+ (LSK) phenotype; F4/80 was used to sort macrophages, Flk1 to mark ECs and Gr-1, known as Ly-6G/Ly-6C, to sort neutrophils. (A) The phospholipases *Pla2g4a* are highly expressed in neutrophils compared to (B-C) *Pla2g4b* and *Pla2g4c*. (D-E) The expression of cyclooxygenases *Cox1* and *Cox2* is enriched in macrophages. (F-H) The prostaglandin synthases *Ptges3* and the prostaglandin

transporters *Slco2b1* specifically expressed in ECs. *Abcc4* was expressed by both ECs and macrophages. (I-N) The prostaglandin receptors *Ptger1*, *Ptger4* are mainly expressed in HSPCs compared to *Ptger2* and *Ptger3*. Statistical analysis was completed using one-way ANOVA, multiple comparison test. *P<.05; **P<.01; ***P<.001; ****P<.0001.

Appendix Table S1. Primer used for quantitative real time PCR of zebrafish expressed genes.

ptger 4a-F	TGCCAATATTTCGGCTTCGTGCTG
ptger 4a-R	ATGCGTAAATGGCGAGTAGGGTGA
ptger1a-F	ACCTGGTGCAATAGTCATGAGGCT
ptger1a-R	AGAAAGAGCGGACACAGTCCGAAA
ptger2a-F	TGCGGATACATCACCATCCCTTGT
ptger2a-R	GTGGCGTAAACATTGGCATACGCT
ptger3-F	TTATTCAGTTGATGGGCATTATGT
ptger3-R	AATTACAGTCCTTCTGCAATTCCT
cox1-F	ACAGTTCCAGTACCAGAACCGCAT
cox1-R	TCCACCAGCTTCTCCAAGCCATAA
cox2a-F	CACTGTTGCCGGACAACTTTCAGA
cox2a-R	TCCAGCAGTCTGTTTGGTGAAGGA
cox2b-F	CTTTACCATTGGCACCCCCT
cox2b-R	CCACCCTTAACACTGCTGGT
ptges3a-F	TATTGGAGGCCATTGACCC
ptges3a-R	GGACAATTCTTCATCCGAGTC
ptges3b-F	CTCAGTGGAGCAGATAATGT
ptges3b-R	GCTCCGTCTAGATCAGGTAA
pla2g4aa-F	CTCTCCCTTCAGCGGCATCA
pla2g4aa-R	GGTCAAGCCACTGTCCACCA
pla2g4ab-F	CATCAATCCAGAGTGGAATGAG
pla2g4ab-R	CCTATTAGGAATGACACCAGC
slco2b1-F	TTGCCCTGCCTCACTTCATT
slco2b1-R	AGGCTGGAGTTGAGTCTGGT
abcc4-F	GTCCGCCTCACCGTCACTC
abcc4-R	CGGCTCTTTCTTCTCCTCCTG

Appendix Table S2. Primer used for quantitative real time PCR of mouse expressed genes.

Mouseq-Pla2g4a-F	CTACGTGCCACCAAAGTAAC
Mouseq-Pla2g4a-R	CCTAGGGTTTCATCCATGAC
Mouseq-Pla2g4b-F	CCCTGTCTGGAATCAGAACT
Mouseq-Pla2g4b-R	CTGATGAGCTGTTCCTCACA
Mouseq-Pla2g4c-F	GCTCCAGTCATTGCTGTCTT
Mouseq-Pla2g4c-R	CTCCAGGCTCTCATGAAAGT
Mouseq-Cox1-F	CCAGTGTGATTGTACTCGCA
Mouseq-Cox1-R	GTAGTCATGCGCTGATGGTG
Mouseq-Cox2-F	CGGACTGGATTCTATGGTGA
Mouseq-Cox2-R	GTGCACATTGTAAGTAGGTGG
Mouseq-Slco2b1-F	CTCAGGACTCACATCAGGAT
Mouseq-Slco2b1-R	GCTGCCGAAATAGCTCACAA
Mouseq-Ptges3-F	CAGCCTGCTTCTGCAAAG
Mouseq-Ptges3-R	GAAGTCCACACTGAGCCA
Mouseq-Abcc4-F	CAGAAGATCGCTCAAAGCAC
Mouseq-Abcc4-R	CTGCACGTGGTAGAAGTACA
Mouseq-Ptger1-F	TTTATTAGCCTTGGGCCTCG
Mouseq-Ptger1-R	TTGCACACTAATGCCGCAAG
Mouseq- Ptger2-F	GAATTGGTGCTCACTGACCT
Mouseq-Ptger2-R	CATCGTGGCCAGACTAAAGA
Mouseq-Ptger3-F	TGTCGGTTGAGCAATGCAAG
Mouseq-Ptger3-R	GCAGAACTTCCGAAGAAGGA
Mouseq-Ptger4-F	CATCTTACTCATCGCCACC
Mouseq Ptger4-R	GCACAGTCTTCCGAAGAAG
