# Supplementary material for: Synergistic prostaglandin E synthesis by myeloid and endothelial cells promotes fetal hematopoietic stem cell expansion in vertebrates
Source: EMBO J. 2022 Aug 4;41(19):e108536. doi: 10.15252/embj.2021108536 (PMC9531293; doi:10.15252/embj.2021108536)
Supplement: Supplementary file 2 — Movie EV1 [file EMBJ-41-e108536-s008.zip › Movie EV1/Movie EV1.docx]

**Movie EV1**.

Time-lapse confocal imaging of the CHT of a cmyb:GFP embryo injected with control morpholino (ctrl-mo). (54-60hpf)
