# Supplementary material for: Synergistic prostaglandin E synthesis by myeloid and endothelial cells promotes fetal hematopoietic stem cell expansion in vertebrates
Source: EMBO J. 2022 Aug 4;41(19):e108536. doi: 10.15252/embj.2021108536 (PMC9531293; doi:10.15252/embj.2021108536)
Supplement: Supplementary file 3 — Movie EV2 [file EMBJ-41-e108536-s004.zip › Movie EV2/Movie EV2.docx]

**Movie EV2**.

Time-lapse confocal imaging of the CHT of a cmyb:GFP embryo injected with slco2b1-morpholino (slco2b1-mo). (54-60hpf)
