# Supplementary material for: Synergistic prostaglandin E synthesis by myeloid and endothelial cells promotes fetal hematopoietic stem cell expansion in vertebrates
Source: EMBO J. 2022 Aug 4;41(19):e108536. doi: 10.15252/embj.2021108536 (PMC9531293; doi:10.15252/embj.2021108536)
Supplement: Supplementary file 12 [file EMBJ-41-e108536-s006.pdf]

THE EMBO JOURNAL

Synergistic prostaglandin E synthesis by myeloid and endothelial cells promotes fetal hematopoietic stem cell expansion in vertebrates

Pietro Cacialli, Marie-Pierre Mailhe, Ingrid Wagner, Doron Merkler, Rachel Golub and Julien Y. Bertrand
DOI: 10.15252/embj.2021108536

Corresponding author: Julien BERTRAND (julien.bertrand@unige.ch)

Review Timeline:

Submission Date:	21st Apr 21
Editorial Decision:	23rd Jun 21
Revision Received:	23rd May 22
Editorial Decision:	11th Jul 22
Revision Received:	13th Jul 22
Accepted:	14th Jul 22

Editor: Daniel Klimmeck

Transaction Report:

(Note: With the exception of the correction of typographical or spelling errors that could be a source of ambiguity, letters and reports are not edited. Depending on transfer agreements, referee reports obtained elsewhere may or may not be included in this compilation. Referee reports are anonymous unless the Referee chooses to sign their reports.)

1st Editorial Decision 23rd Jun 2021

Dear Dr Bertrand,

Thank you again for the submission of your manuscript (EMBOJ-2021-108536) to The EMBO Journal and in addition providing us with a preliminary revision plan. As mentioned earlier, Your study has been sent to three reviewers for evaluation. However, one referee got much delayed and, in the end, did not send us his-her report. In the interested of the timeliness of the data, we now decided to base our decision on the two other reports, which I enclose below.

As you will see, the referees acknowledge the quality of the analyses and potential interest of your findings. However, although they also express major concerns. In more detail, the referees state that additional experiments are required to substantiate the zebrafish analysis and tissue relevance of the intercellular cooperation model proposed (Ref#1, pts.1,5; ref#3, pt.3). In addition, they point to incomplete methods characterization for the cd45 promoter model (ref#3, pt.1) and state that the conservation of the stromal cell cooperation in the mouse is not comprehensively supported by the data (ref#1, pt.8; ref#3, pt.2).

Given the interest stated and broader angle of your findings, we are overall able to invite you to revise your manuscript experimentally to address the referees' comments, along the lines sketched in your outline. I need to stress though that we do require strong support from the referees on a revised version of the study in order to move on to publication of the work. As to the open outcome of the revisional work I suggest keeping EMBO Reports in mind for this study as an alternative venue.

Please feel free to contact me if you have any questions or need further input on the referee comments.

We generally allow three months as standard revision time. As a matter of policy, competing manuscripts published during this period will not negatively impact on our assessment of the conceptual advance presented by your study. However, we request that you contact the editor as soon as possible upon publication of any related work, to discuss how to proceed. Should you foresee a problem in meeting this three-month deadline, please let us know in advance and we may be able to grant an extension.

When submitting your revised manuscript, please carefully review the instructions below and include the following items:

1) a .docx formatted version of the manuscript text (including legends for main figures, EV figures and tables). Please make sure that the changes are highlighted to be clearly visible.

2) individual production quality figure files as .eps, .tif, .jpg (one file per figure).

3) a .docx formatted letter INCLUDING the reviewers' reports and your detailed point-by-point response to their comments. As part of the EMBO Press transparent editorial process, the point-by-point response is part of the Review Process File (RPF), which will be published alongside your paper.

4) a complete author checklist, which you can download from our author guidelines (https://wol-prod-cdn.literatumonline.com/pb-assets/embo-site/Author Checklist%20-%20EMBO%20J-1561436015657.xlsx). Please insert information in the checklist that is also reflected in the manuscript. The completed author checklist will also be part of the RPF.

5) Please note that all corresponding authors are required to supply an ORCID ID for their name upon submission of a revised manuscript.

6) It is mandatory to include a 'Data Availability' section after the Materials and Methods. Before submitting your revision, primary datasets produced in this study need to be deposited in an appropriate public database, and the accession numbers and database listed under 'Data Availability'. Please remember to provide a reviewer password if the datasets are not yet public (see https://www.embopress.org/page/journal/14602075/authorguide#datadeposition).
In case you have no data that requires deposition in a public database, please state so in this section. Note that the Data Availability Section is restricted to new primary data that are part of this study.
*** Note - All links should resolve to a page where the data can be accessed. ***

7) Our journal encourages inclusion of *data citations in the reference list* to directly cite datasets that were re-used and obtained from public databases. Data citations in the article text are distinct from normal bibliographical citations and should directly link to the database records from which the data can be accessed. In the main text, data citations are formatted as follows: "Data ref: Smith et al, 2001" or "Data ref: NCBI Sequence Read Archive PRJNA342805, 2017". In the Reference list, data citations must be labeled with "[DATASET]". A data reference must provide the database name, accession number/identifiers and a resolvable link to the landing page from which the data can be accessed at the end of the reference. Further instructions are available at .

8) We would also encourage you to include the source data for figure panels that show essential data. Numerical data can be provided as individual .xls or .csv files (including a tab describing the data). For 'blots' or microscopy, uncropped images should

be submitted (using a zip archive or a single pdf per main figure if multiple images need to be supplied for one panel). Additional information on source data and instruction on how to label the files are available at .

9) We replaced Supplementary Information with Expanded View (EV) Figures and Tables that are collapsible/expandable online (see examples in https://www.embopress.org/doi/10.15252/embj.201695874). A maximum of 5 EV Figures can be typeset. EV Figures should be cited as 'Figure EV1, Figure EV2" etc. in the text and their respective legends should be included in the main text after the legends of regular figures.

- For the figures that you do NOT wish to display as Expanded View figures, they should be bundled together with their legends in a single PDF file called *Appendix*, which should start with a short Table of Content. Appendix figures should be referred to in the main text as: "Appendix Figure S1, Appendix Figure S2" etc. See detailed instructions regarding expanded view here: .

- Additional Tables/Datasets should be labelled and referred to as Table EV1, Dataset EV1, etc. Legends have to be provided in a separate tab in case of .xls files. Alternatively, the legend can be supplied as a separate text file (README) and zipped together with the Table/Dataset file.

10) When assembling figures, please refer to our figure preparation guideline in order to ensure proper formatting and readability in print as well as on screen:
http://bit.ly/EMBOPressFigurePreparationGuideline

Please remember: Digital image enhancement is acceptable practice, as long as it accurately represents the original data and conforms to community standards. If a figure has been subjected to significant electronic manipulation, this must be noted in the figure legend or in the 'Materials and Methods' section. The editors reserve the right to request original versions of figures and the original images that were used to assemble the figure.

11) For data quantification: please specify the name of the statistical test used to generate error bars and P values, the number (n) of independent experiments (specify technical or biological replicates) underlying each data point and the test used to calculate p-values in each figure legend. The figure legends should contain a basic description of n, P and the test applied. Graphs must include a description of the bars and the error bars (s.d., s.e.m.).

Thank you for the opportunity to consider your work for publication.
I look forward to your revision.

Kind regards,

Daniel Klimmeck

Daniel Klimmeck, PhD
Senior Editor
The EMBO Journal

Additional Instructions for preparing your revised manuscript:

Please make sure you upload a letter of response to the referees' comments together with the revised manuscript.

Please also check that the title and abstract of the manuscript are brief, yet explicit, even to non-specialists.

Please remember: Digital image enhancement is acceptable practice, as long as it accurately represents the original data and conforms to community standards. If a figure has been subjected to significant electronic manipulation, this must be noted in the figure legend or in the 'Materials and Methods' section. The editors reserve the right to request original versions of figures and the original images that were used to assemble the figure.

Further information is available in our Guide For Authors: https://www.embopress.org/page/journal/14602075/authorguide

Revision to The EMBO Journal should be submitted online within 90 days, unless an extension has been requested and

approved by the editor; please click on the link below to submit the revision online before 21st Sep 2021:

Link Not Available

Referee #1:

In this manuscript Caciali et al. examine the role of PGE2 in the expansion of HSPCs in the caudal hematopoietic tissue of zebrafish. As expected, they find that PGE2 is important for the proliferation of HSCs in CHT. However, they fully dissect the biosynthetic pathway of PGE2 and surprisingly they find that three different CHT cell types are involved in the biosynthesis of PGE2. Neutrophils produce arachidonic acid; myeloid cells are responsible for the production of PGH2 which is the precursor of PGE2 and endothelial cells produce PGE2 which is later used by HSPCs. They verified this model by identifying the expression of enzymes responsible for each part of the PGE2 biosynthetic pathway at the respective cell types and by ablation and rescue experiments. Finally, they showed that similar cell types in the mouse fetal liver express similar enzymes and thus the partitioning of the biosynthetic pathway of PGE2 is conserved in mammals.
Overall, this is an interesting and novel manuscript but some points need to be addressed:
Major points:
1. The authors should use publicly available single cell RNA seq data for the CHT and the fetal liver in zebrafish and mouse respectively and check the expression of the PGE2 biosynthetic enzymes in different cell types. In this way they will strengthen their conclusions and examine the heterogeneity of expression within specific cell types. It would also be interesting to check respective human data.
2. It is interesting but also unexpected the fact that one biosynthetic pathway is distributed in different cell types and this feature is "evolutionary" conserved. Can the authors include in the discussion their thoughts about the necessity of such a distribution?
3. Can the authors use a labeled form of PGH2 and trace its "absorbance" from the endothelial cells?
4. Can the authors show that the mutation on the slco2b1 gene leads to absence of the protein or degraded mRNA?
5. Can the authors show by double in situ that expression of slcob1 is indeed present in endothelial cells?
6. In the rescue experiments, for example with PGH2 or PGE2 the authors should clearly state and comment on the effect of the drugs on the control embryos. Ideally a dose that does not affect the controls should be chosen. Otherwise, it is difficult to interpret these experiments. That being said most of the experiments include these controls and the effects on control embryos are not major.
7. The authors show that specific overexpression of slcob1 rescues the phenotype only if expressed in endothelial but not in myeloid cells. However small amounts of ptgers3a/3b are expressed in myeloid cells. I would have expected that some partial rescue should be feasible. Can the authors explain
8. The authors show that the expression of the respective enzymes is conserved in fetal liver populations. However, it would be commendable if they could perform some functional experiments to verify their model.

Minor points:

1. In Figure S4 the authors perform qPCR analysis but it is not clear in which population. Can the authors mention this information? It would be preferable to perform qPCR in sorted populations as in Figure 1.
2. It would be helpful to put the number of the Figure in each figure
3. In Figure 5d,e the respective figure legend is missing
4. Can the authors include the biosynthetic pathway of PGE2 in their model figure?

Referee #3:

In this manuscript titled "Myeloid and endothelial cells cooperate to promote hematopoietic stem cells expansion in the fetal niche," Cacialli and colleagues describe a series of experiments that investigate the cells involved in the Prostaglandin E (PGE2) synthesis pathway within the caudal hematopoietic tissue (CHT) in zebrafish, a major developmental hematopoietic niche. They demonstrated that the disrupting production of PGE2 diminishes HSPC expansion within the CHT. Their data suggest that PGE2 production is orchestrated by a complex handoff of precursors and intermediates among myeloid cells, which normally produce PGH2/PGG2, endothelial cells (ECs), which uptake these precursors via Slco2b1 and convert them into PGE2. While the data are generally compelling, there are a few major concerns that need to be further addressed.

1. The authors have generated a new conditional hematopoietic cell depletion system based on expression of NTR under the cd45 promoter. The line is incompletely characterized, and thus difficult to fully interpret the findings from the experiments with the transgenics. Although they showed that cd45:CFP-NTR minimally overlapped to runx1:mcherry+ HSPCs at 72 hpf, they did not show the expression at 48 hpf, which is the starting point of the metronidazole (MTZ) treatment. Also, they fail to demonstrate which myeloid cells express the cd45:CFP-NTR transgene and which ones are depleted during the 48-72 hpf developmental window. This can be accomplished through the analysis of a secondary marker (i.e. transgenics marking

granulocytes [lyz, mpx], macrophages [mfap4, mpeg]) along with cd45:CFP-NTR. Lastly in terms of cd45:CFP-NTR, it does not appear that MTZ treatment alone was tested on non-transgenic animals. As MTZ is a potent antibiotic, it would be helpful to see whether treatment for their particular time frame was harmful to their animals or played a role in decreased HSPC expansion.

2. Taking past work into account and the results from this manuscript, there is a solid case for the interaction between certain niche cells (ie macrophages, neutrophils, ECs) in the pathway of PGE2 synthesis in developing zebrafish. However, the prostaglandin pathway expression patterns described in mouse FL cells does not fully mimic that shown in the zebrafish. For example, the expression of Cox1 is found in both macrophages and Flk1 ECs, Ptges3 is expressed to similar levels in ECs and neutrophils, and Abcc4 is expressed similarly in ECs and macrophages. These data suggests that the expression of PGE2 synthesis components are not as restricted in mouse FL compared to zebrafish. This suggests that the precursor/intermediate handoff might not be conserved. Functional assays would need to be performed to make this claim.

3. For the PGE2 biosynthetic hand-off model, the cells expressing the components must be in close proximity. In situ hybridization, immunohistochemistry, or immunofluorescence showing the expression of critical components with zebrafish CHT or murine FL should be provided to support the model.

Minor points
a. The authors showed strong evidence that slco2b1 deficiency leads to a decrease in proliferating HSPCs (with their pH3 immunostaining) and subsequent expansion (time-lapse microscopy). An apoptosis assay such as with caspase or Annexin/PI assays would determine if the HSPCs in the CHT are they dying off.
b. As a minor point, the flow plot gating strategy they used to derive this final flow plot in Suppl Fig 2B would be helpful for future readers trying to recreate their experiment and use their new system for myeloid depletion.
c. In addition, Figure 6A and 6B legend needs to include an explanation regarding the symbols (+, ++, +++,++++,-) and colors used.
d. This statement in the introduction seems like an overstatement and should be modified. "While in vitro culture systems have allowed a wide comprehension of key signalling involved in HSC differentiation, the mechanisms controlling HSC expansion, which only occurs during embryogenesis ..." HSC expansion can occur in adults in response to regenerative signals, thus the use of "only" is incorrect.
e. The tissue-specific slco2b1 rescue experiment is an important component to demonstrate the selective importantce of this transporter in ECs versus other cells. These data should be moved to the main figures.

We would like to thank the two reviewers who carefully read our manuscript. And we also apologize for the time it took us to complete our revision. As you can read below, we have carefully addressed all major and minor points according to the plan that was established and validated after the first round of revision.

Referee #1:

In this manuscript Cacialli et al. examine the role of PGE2 in the expansion of HSPCs in the caudal hematopoietic tissue of zebrafish. As expected, they find that PGE2 is important for the proliferation of HSCs in CHT. However, they fully dissect the biosynthetic pathway of PGE2 and surprisingly they find that three different CHT cell types are involved in the biosynthesis of PGE2. Neutrophils produce arachidonic acid; myeloid cells are responsible for the production of PGH2 which is the precursor of PGE2 and endothelial cells produce PGE2 which is later used by HSPCs. They verified this model by identifying the expression of enzymes responsible for each part of the PGE2 biosynthetic pathway at the respective cell types and by ablation and rescue experiments. Finally, they showed that similar cell types in the mouse fetal liver express similar enzymes and thus the partitioning of the biosynthetic pathway of PGE2 is conserved in mammals.

Overall, this is an interesting and novel manuscript but some points need to be addressed:

Major points:

1. The authors should use publicly available single cell RNA seq data for the CHT and the fetal liver in zebrafish and mouse respectively and check the expression of the PGE2 biosynthetic enzymes in different cell types. In this way they will strengthen their conclusions and examine the heterogeneity of expression within specific cell types. It would also be interesting to check respective human data.

We have followed this advice and spent much time (and a lot of money) reanalyzing the zebrafish CHT set of data (GSE120581) from Feng Liu's lab (Xue et al, Cell Rep 2019; Xia et al, PNAS 2021). We are sorry to say that this analysis was almost completely unfruitful, because despite what has been published by this group, we could not find any cluster corresponding to endothelial cells (by doing a totally unbiased clustering analysis). We asked this group directly if they had forgotten to add a data set in their publicly available dataset, but it turned out that it was not the case. Explaining our issue with their data, they actually explained the following: "First, it is true that we also noticed that some endothelial-associated transcripts, such as kdrl and tie2, were expressed at low levels. We think that the reason why there were a few endothelial genes in our scRNA-seq data is due to that a part of endothelial transcripts expressed at a lower level were filtered out as sample noise. Then for cell annotation, since 50,000 kdrl:mCherry+ cells, 50,000 CD41:GFP+ cells and 50,000 double negative cells were mixed together for 10 × Genomics analysis, we used step by step method to do cell type annotation. Manual annotation was performed iteratively, which included validating proposed cell labels with known markers and further investigating clusters for which the gene signatures indicated additional diversity (1). Cluster 2 and 3 in our work were annotated as endothelial cells mainly based on the expression of fli1a, fli1b and gata2b, which are known

as key TFs in endothelial or hemogenic endothelial cells.” According to our bioinformatician and to our own knowledge, it therefore appears that there were never any endothelial cells in this analysis, as what they annotated as endothelial cells mostly appears as thrombocytes to us. One will draw their own conclusions concerning the two sus-mentionned papers.

Concerning the mouse fetal liver, we directly asked the authors from this paper - Wang et al, Cell Research 2020 (Cheng-Ran Xu lab) – to dig out the expression of all our genes of interest in their different subsets (E14.5 Fetal Livers). They kindly and rapidly sent us violin plots, and from that we could only get that Ptgs1 (Cox1) was highly expressed by macrophages, and that ptges3 was expressed by all subsets (macrophages, neutrophils, endothelial cells, HSPCs). The expression of all other genes was undetectable (pla2g4a, pla2g4b, pla2g4c, ptgs2/cox2, slco2b1, abcc4, ptger1-4). Therefore, we decide to not include these results as they were really incomplete, which was making no sense for our manuscript. Disappointing but this kind of experience is inherent to the scRNAseq techniques where not all transcripts can be detected in each single cells.

Concerning human data, we could not find any data set that would be easy to reanalyze in a most efficient way.

Therefore, we stick to our quantitative PCR analysis, which to our opinion is much more reliable, as there is no bias.

2. It is interesting but also unexpected the fact that one biosynthetic pathway is distributed in different cell types and this feature is "evolutionary" conserved. Can the authors include in the discussion their thoughts about the necessity of such a distribution?

This question has really occupied our minds for some time, but it appears that there are other examples in our environment and even in our own organisms. We have discussed this point, and added many examples of now distributed metabolic pathways, such as the biosynthesis of testosterone in the fetal testis, estrogens in the ovary, Vitamin D and even opioids in the poppy. Whether or not this allows a certain level of regulation is another question.

3. Can the authors use a labeled form of PGH2 and trace its "absorbance" from the endothelial cells?

As we use commercial PGH2, it appears that there is no way to conjugate this with a fluorescent probe, considering the opinion of our lipid expert here at the University of Geneva (Pr. Anne-Claude Gavin).

We used also NBD-AA, (company Avanti Polar Lipids), a fluorescently labelled arachidonic acid (coupled to NBD). But unfortunately, it was technically not possible to detect AA in zebrafish, as all cells would incorporate it.

4. Can the authors show that the mutation on the slco2b1 gene leads to absence of the protein or degraded mRNA?

it is indeed impossible to amplify this *slco2b1* mRNA from mutant embryos. This result is now integrated in the new version of the manuscript, Suppl. Fig.9b.

5. Can the authors show by double in situ that expression of slcob1 is indeed present in endothelial cells?

The ISH has been done and corroborates our qPCR data. The signals for *slco2b1* and *kdrl* do overlap in the venous part of the CHT at 48hpf (Suppl. Fig.1b).

6. In the rescue experiments, for example with PGH2 or PGE2 the authors should clearly state and comment on the effect of the drugs on the control embryos. Ideally a dose that does not affect the controls should be chosen. Otherwise, it is difficult to interpret these experiments. That being said most of the experiments include these controls and the effects on control embryos are not major.

Thank you for noting that we showed convincing controls for most of the experiments, and we apologize if we missed this for one set of data. We are not really sure which figure the reviewer is referring to. In figure 2, we show PGH2 and PGE2 treatments on non-MTZ-treated, which does not affect embryonic development (morphology was OK) and therefore slightly augments the numbers of HSPCs (runx1+) and myeloid cells (CD45CFP+). In figure 4, we also treat control morphants with PGE2 and again no developmental effect and a small increase of HSPCs (cmyb:GFP^{+}). The reviewer is probably talking about Figure 5… We did not added the PGH2 treatment on non-MTZ-treated embryos as it was the same control as for Figure 2. One can compare the numbers with Figure 2, if needed, as all these experiments were done at the same time.

7. The authors show that specific overexpression of slco2b1 rescues the phenotype only if expressed in endothelial but not in myeloid cells. However small amounts of ptges3a/3b are expressed in myeloid cells. I would have expected that some partial rescue should be feasible. Can the authors explain

Indeed, there is some expression of the PGE2 synthetase in other cells, which could therefore produce some small quantities of PGE2. However, it is well established that PGE2 is released in the extracellular milieu through Abcc4, which is hardly to not expressed at all by myeloid cells. Therefore, even if myeloid cells could produce some PGE2, they could not release it towards HSPCs.

8. The authors show that the expression of the respective enzymes is conserved in fetal liver populations. However, it would be commendable if they could perform some functional experiments to verify their model.

We would have loved to perform functional data, but unfortunately this would mean using a lot of CRE/lox mice, if the idea was to delete specifically an enzyme or a transporter in a

specific cell type. As for in vitro experiments, they would be difficult to realize as many other cell types could be important for HSPCs expansion in the fetal liver, compared to the CHT. However, we hope that our work will inspire mouse developmental hematologists. Somehow, an important functional data has been published in the mean time showing the role of macrophages. Feng Liu's group has shown that depletion of macrophages during fetal life in mouse could decrease the number of HSCs in the fetal liver (Gao et al., Cell Research 2022). This aspect has been added to the discussion. We had already shown that HSPCs were located close to macrophages in the human fetal liver (Cacialli et al, 2021), and we have now recapitulated this data in the mouse fetal liver (figure 8). We also show that HSPCs (cKit high) are located close to the vasculature at E13 and further away at E16, confirming previously published data from the Frenette lab (Kahn et al., Science 2016). As for neutrophils, their numbers are really low at E13, which resulted in a long distance between them and HSPCs at E13. As their number increases with development, HSPCs are getting closer by E16.

Minor points:

1. In Figure S4 the authors perform qPCR analysis but it is not clear in which population. Can the authors mention this information? It would be preferable to perform qPCR in sorted populations as in Figure 1.

The Suppl. Fig. 4 in the new version of the manuscript is the Suppl. Fig. 6, we have performed qPCR on dissected CHT to show that the loss of myeloid cells led to the specific loss of the enzymes specifically expressed by myeloid cells, and that this loss was not compensated by other cell types. As we deplete totally myeloid cells, it is therefore not possible to sort these cells to show that they do not express these enzymes. Moreover, in these MTZ-treated CD45:CFP-NTR transgenic embryos, the ablation of myeloid cells leads to a significant decreased of HSPCs, which makes it also difficult to sort these cells.

2. It would be helpful to put the number of the Figure in each figure

We added the number of the Figure in each of them.

3. In Figure 5d,e the respective figure legend is missing

Apologies, we added it.

4. Can the authors include the biosynthetic pathway of PGE2 in their model figure?

Excellent graphic idea, thanks. We changed the whole model as a liner model where all cell types follow each other from left to right, therefore allowing to "paste" the biochemical synthesis pathway.

Referee #3:

In this manuscript titled "Myeloid and endothelial cells cooperate to promote hematopoietic

stem cells expansion in the fetal niche," Cacialli and colleagues describe a series of experiments that investigate the cells involved in the Prostaglandin E (PGE2) synthesis pathway within the caudal hematopoietic tissue (CHT) in zebrafish, a major developmental hematopoietic niche. They demonstrated that the disrupting production of PGE2 diminishes HSPC expansion within the CHT. Their data suggest that PGE2 production is orchestrated by a complex handoff of precursors and intermediates among myeloid cells, which normally produce PGH2/PGG2, endothelial cells (ECs), which uptake these precursors via Slco2b1 and convert them into PGE2. While the data are generally compelling, there are a few major concerns that need to be further addressed.

1. The authors have generated a new conditional hematopoietic cell depletion system based on expression of NTR under the cd45 promoter. The line is incompletely characterized, and thus difficult to fully interpret the findings from the experiments with the transgenics. Although they showed that cd45:CFP-NTR minimally overlapped to runx1:mcherry+ HSPCs at 72 hpf, they did not show the expression at 48 hpf, which is the starting point of the metronidazole (MTZ) treatment. Also, they fail to demonstrate which myeloid cells express the cd45:CFP-NTR transgene and which ones are depleted during the 48-72 hpf developmental window. This can be accomplished through the analysis of a secondary marker (i.e. transgenics marking granulocytes [lyz, mpx], macrophages [mfap4, mpeg]) along with cd45:CFP-NTR. Lastly in terms of cd45:CFP-NTR, it does not appear that MTZ treatment alone was tested on non-transgenic animals. As MTZ is a potent antibiotic, it would be helpful to see whether treatment for their particular time frame was harmful to their animals or played a role in decreased HSPC expansion.

We apologize for this. The ptprc/cd45 promoter is the exact same that was used to generate the CD45:DsRED transgenic line which was fully characterized before (Wittamer, Bertrand et al, Blood 2011). It is true that identical promoters can behave differently when used in transgenesis, therefore we proceeded to a basic characterization as required.

First, we analyzed triple transgenic (cd45:CFP-NTR;mpeg1:mCherry;mpx:GFP) at 60hpf by live-imaging (Suppl.Fig.2b). This data shows that all CFP overlap with mCherry or GFP positive cells, confirming that our cd45 promoter is active in macrophage and neutrophil cells.

Next, we analyzed double transgenic (cd45:CFP-NTR;runx1:mCherry) embryos at 48 and 72hpf by cytometry, and triple transgenic (cd45:CFP-NTR;runx1:mCherry;mpeg1:GFP) at 60hpf by live-imaging (Suppl.Fig.3a-b-c). Our data clearly show that CFP and mCherry do not overlap, meaning that the 7.6kb cd45 promoter is inactive in embryonic HSPCs.

As previously reported, the ablation system allows killing of NTR-expressing cells upon addition of metronidazole (MTZ). Importantly, expression alone or administration of MTZ to non-transgenic embryos does not induce apoptosis. By contrast, a single treatment with MTZ for 24h was sufficient to ablate myeloid cells in cd45:CFP-NTR transgenic embryos. We show the ablation of macrophage and neutrophil by in situ hybridization for *mfap4* and *mpx* after 24h of MTZ treatment in cd45:CFP-NTR transgenic embryos (Suppl.Fig.4a-b).

Finally, we have performed MTZ treatments of wild-type AB* embryos, and then *cmyb* ISH. None of the MTZ doses, from 1mM to 10 mM affected the morphology nor the HSPC content (*cmyb* signal) at 72hpf (Suppl. Fig. 7a).

2. Taking past work into account and the results from this manuscript, there is a solid case for the interaction between certain niche cells (ie macrophages, neutrophils, ECs) in the pathway of PGE2 synthesis in developing zebrafish. However, the prostaglandin pathway expression patterns described in mouse FL cells does not fully mimic that shown in the zebrafish. For example, the expression of Cox1 is found in both macrophages and Flk1 ECs, Ptges3 is expressed to similar levels in ECs and neutrophils, and Abcc4 is expressed similarly in ECs and macrophages. This data suggests that the expression of PGE2 synthesis components are not as restricted in mouse FL compared to zebrafish. This suggests that the precursor/intermediate handoff might not be conserved. Functional assays would need to be performed to make this claim.

As mentioned to the other reviewer, it will be very difficult to perform functional assays, either in vivo or in vitro. Therefore, we will re-assess and tone down our claim concerning this point. Again, evolution has probably added many layers of redundancy and shifting from the zebrafish CHT to the liver in mammals has also added the contribution of many other cell types (such as hepatocytes).

3. For the PGE2 biosynthetic hand-off model, the cells expressing the components must be in close proximity. In situ hybridization, immunohistochemistry, or immunofluorescence showing the expression of critical components with zebrafish CHT or murine FL should be provided to support the model.

We already know that HSPCs and ECs are in close contact (Tamplin, 2015, Mahony, 2016). In the new version of the manuscript is the Suppl. Fig.3b we show that macrophages and neutrophils are in the vicinity of HSCs by using the triple transgenic *(runx1:mCherry; CD45:CFP; mpeg1:GFP*), where HSPCs = mCherry (red); macrophages = GFP + CFP (cyan); and neutrophils = CFP (blue).

Concerning the mouse, it was also shown that HSPCs are in close proximity with the FL vasculature (Tamplin, Cell 2015; Khan et al. Science 2016).
We have previously shown that human HSPCs are located in close proximity to macrophages in the human fetal liver (Cacialli et al, Nature Communications, 2021). New data from the Liu lab has also recently shown this close association between HSPCs and macrophages in the mouse fetal liver (Gao et al, Cell research 2022). Moreover, they performed macrophage depletion assays to show the negative impact on HSPC expansion in the fetal liver. These points have been added to the discussion.

We have used mouse FL at E13 and E16 to perform antibody stainings against ECs, macrophages, neutrophils and HSPCs (see figure 8). This allowed to visualize close association between HSPCs, vessels and macrophages at E13, which was lost at E16. Neutrophils were not close to HSPCs at E13. They are very difficult to observe as their numbers are low in the E13 fetal liver. However at later stages, the distance between HSPCs and neutrophils is smaller as there are a lot more neutrophils in the fetal liver. However, as pointed in the discussion,

cells do not necessarily need to be in close proximity to collaborate in a metabolic pathway. For example it takes different organs to synthetize the active form of Vitmain D (Skin, liver and kidney).

Minor points

a. The authors showed strong evidence that slco2b1 deficiency leads to a decrease in proliferating HSPCs (with their pH3 immunostaining) and subsequent expansion (time-lapse microscopy). An apoptosis assay such as with caspase or Annexin/PI assays would determine if the HSPCs in the CHT are they dying off.

We have assessed apoptosis assay by acridine orange stainings, and could not observe much, as seen in Suppl. Fig. 12a-b. Moreover, the number of cells does not decrease dramatically, but rather stays unchanged during our time-lapse analyses (Suppl. videos 1-2). Therefore, the lack of PGE2 signaling might not kill HSPCs but rather prevent them from actively proliferating. Indeed we found a significative decrease of pH3 marker in cmyb:GFP positive cells of slco2b1-morphants (Fig. 5a-b).

b. As a minor point, the flow plot gating strategy they used to derive this final flow plot in Suppl Fig 2B would be helpful for future readers trying to recreate their experiment and use their new system for myeloid depletion.

We now show the gating strategy, in the new version of the manuscript is the Suppl. Figure 3a.

c. In addition, Figure 6A and 6B legend needs to include an explanation regarding the symbols (+, ++, +++,++++,-) and colors used.

In the revised manuscript, these are now Figures 7a and 7b.
The different symbols correspond to the Ct value to which the threshold of detection was applied during the analysis of the qPCR.

very high expression (++++)	Ct <24
high expression (+++)	25<Ct<28
medium expression (++)	29<Ct<32
low expression (+)	33<Ct<36
very low to no expression (-)	Ct>37.

The color gradients correspond to the levels of expression detected. As for colors themselves, they were chosen arbitrarily, but they correspond to distinct biosynthetic steps occurring in distinct cell subsets, which harbor the same color in panels c and d.

d. This statement in the introduction seems like an overstatement and should be modified. "While in vitro culture systems have allowed a wide comprehension of key signalling involved in HSC differentiation, the mechanisms controlling HSC expansion, which only occurs during embryogenesis ..." HSC expansion can occur in adults in response to regenerative signals, thus the use of "only" is incorrect.

We meant that in steady state, HSCs only expand or mainly expand during fetal life. We have modified this statement.

e. The tissue-specific slco2b1 rescue experiment is an important component to demonstrate the selective importance of this transporter in ECs versus other cells. These data should be moved to the main figures.

Thanks for appreciating this. In the new version of the manuscript these data are present in the Figure 6.

1st Revision - Editorial Decision 11th Jul 2022

Dear Dr Julien Bertrand,

Thank you for submitting your revised manuscript (EMBOJ-2021-108536R) to The EMBO Journal. Your amended study was sent back to the two referees for their re-evaluation, and we have received comments from both of them, which I enclose below. As you will see, the reviewers stated that their concerns have been comprehensively resolved and they are now in favour of publication, pending minor revision.

Thus, we are pleased to inform you that your manuscript has been accepted in principle for publication in The EMBO Journal.

Please carefully consider the remaining point of referee #3 by revising the discussion of the findings and claims made where appropriate.

Further, we now need you to take care of a number of minor issues related to formatting and data presentation as detailed below, which should be addressed at re-submission.

Please contact me at any time if you have additional questions related to below points.

As you might have noted on our web page, every paper at the EMBO Journal now includes a 'Synopsis', displayed on the html and freely accessible to all readers. The synopsis includes a 'model' figure as well as 2-5 one-short-sentence bullet points that summarize the article. I would appreciate if you could provide this figure and the bullet points.

Thank you for giving us the chance to consider your manuscript for The EMBO Journal. I look forward to your final revision.

Again, please contact me at any time if you need any help or have further questions.

Kind regards,

Daniel Klimmeck

Daniel Klimmeck PhD
Senior Editor
The EMBO Journal

Formatting changes required for the revised version of the manuscript:

>> Please provide maximally five keywords for the manuscript.

>> Adjust the title of the 'Competing Interests' section to 'Disclosure and Competing Interests Statement'.

>> Author Contributions: Please remove the author contributions information from the manuscript text. Note that CRediT has replaced the traditional author contributions section as of now because it offers a systematic machine-readable author contributions format that allows for more effective research assessment. and use the free text boxes beneath each contributing author's name to add specific details on the author's contribution.
More information is available in our guide to authors.
https://www.embopress.org/page/journal/14602075/authorguide

>> Dataset EV legends: The two movies need their legends removed from the suppl. information file and zipped with the corresponding movie file. Rename "Movie EV1" and 2. and add titles & legends to the files.

>> Appendix file: please merge the current suppl. information file with suppl. figures. Rename figures "Appendix Figure S1" etc and tables "Appendix Table S1" etc. Add a ToC on its first page and remove movie legends.

>> The Figures should be uploaded individually as high-resolution files using the file type 'Figure'.

>> Make sure to indicate label "D" in Figure 7.

>> Callouts: Add callouts for Movies EV1, EV2, and Figure panels 8E,F.

>> Reference format: please adjust to EMBO Journal style, listing ten authors before et al. .

>> Dataset availability: please change the title to 'Dataset availability section' and make the repository freely accessible.

>> Please consider additional changes and comments from our production team as indicated by the .doc file enclosed and leave changes in track mode.

Further information is available in our Guide For Authors: https://www.embopress.org/page/journal/14602075/authorguide

Please use the link below to submit your revision:

Link Not Available

Referee #1:

The authors have done their best to answer the reviewer questions. I have no further comments

Referee #3:

The reviewers sufficiently addressed most concerns. The claim of conservation of the triad remains incomplete though. As stated by the authors, conditional knockout mouse models for each cell type is beyond the scope of this paper. Instead, the authors need to soften the concluding sentence. While the evidence mentioned in the discussion clearly point to an important role for macrophages and neutrophils in HSPC biology, it could be independent on PGE2 synthesis. The last sentence should be modified along the lines of the following statements.

Our zebrafish studies strongly point to the existence of a triad, composed of neutrophils, macrophages and ECs, that controls the
fate of HSPCs. Our expression analysis of PGE2 biosynthetic components within the fetal liver along with the evidence that macrophages and neutrophils play a role in HSPC biology, suggests that such a handoff mechanism for PGE2 production and regulation of HSPCs is likely conserved across vertebrates.

The authors performed the requested editorial changes.

2nd Revision - Editorial Decision 14th Jul 2022

Dear Dr Julien Bertrand,

Thank you for submitting the revised version of your manuscript. I have now evaluated your amended manuscript and concluded that the remaining minor concerns have been sufficiently addressed.

Thus, I am pleased to inform you that your manuscript has been accepted for publication in the EMBO Journal.

Please note that it is EMBO Journal policy for the transcript of the editorial process (containing referee reports and your response letter) to be published as an online supplement to each paper.

Also, in case you might NOT want the transparent process file published at all, you will also need to inform us via email immediately. More information is available here:
https://www.embopress.org/page/journal/14602075/authorguide#transparentprocess

----

Please note that in order to be able to start the production process, our publisher will need and contact you shortly regarding the page charge authorisation and licence to publish forms.

Authors of accepted peer-reviewed original research articles may choose to pay a fee in order for their published article to be made freely accessible to all online immediately upon publication. The EMBO Open fee is fixed at $6,100 USD / £4,950 GBP / €5,500 EUR (+ VAT where applicable).

We offer two licenses for Open Access papers, CC-BY and CC-BY-NC-ND.
For more information on these licenses, please visit:
http://creativecommons.org/licenses/by-nc-nd/3.0/deed.en_US

Notably, please be reminded that under the DEAL agreement of European scientific institutions with our publisher Wiley, you could be eligible for free publication of your article in the open access format. Please contact either the administration at your institution or Wiley (embojournal@wiley.com) to clarify further questions.

Should you be planning a Press Release on your article, please get in contact with embojournal@wiley.com as early as possible, in order to coordinate publication and release dates.

On a different note, I would like to alert you that EMBO Press is currently developing a new format for a video-synopsis of work published with us, which essentially is a short, author-generated film explaining the core findings in hand drawings, and, as we believe, can be very useful to increase visibility of the work. This has proven to offer a nice opportunity for exposure i.p. for the first author(s) of the study. Please see the following link for representative examples and their integration into the article web page:
https://www.embopress.org/video_synopses
https://www.embopress.org/doi/full/10.15252/embj.2019103932

Please let me know, should you be interested to engage in commissioning a similar video synopsis for your work. According operation instructions are available and intuitive.

Finally, we have noted that the submitted version of your article is also posted on the preprint platform bioRxiv. We would appreciate if you could alert bioRxiv on the acceptance of this manuscript at The EMBO Journal in order to allow for an update of the entry status. Thank you in advance!

If you have any questions, please do not hesitate to call or email the Editorial Office.

Thank you for this contribution to The EMBO Journal and congratulations on a successful publication!

Please consider us again in the future for your most exciting work.

Kind regards,

Daniel Klimmeck

Daniel Klimmeck, PhD
Senior Editor
The EMBO Journal
EMBO
Postfach 1022-40
Meyerhofstrasse 1
D-69117 Heidelberg
contact@embojournal.org
Submit at: http://emboj.msubmit.net

EMBO Press Author Checklist

Corresponding Author Name: Pr JULIEN Y. BERTRAND
Journal Submitted to: EMBO Journal
Manuscript Number: EMBOJ-2021-108536R

USEFUL LINKS FOR COMPLETING THIS FORM
The EMBO Journal - Author Guidelines
EMBO Reports - Author Guidelines
Molecular Systems Biology - Author Guidelines
EMBO Molecular Medicine - Author Guidelines

Reporting Checklist for Life Science Articles (updated January 2022)
This checklist is adapted from Materials Design Analysis Reporting (MDAR) Checklist for Authors. MDAR establishes a minimum set of requirements in transparent reporting in the life sciences (see Statement of Task: 10.31222/osf.io/9sm4x). Please follow the journal's guidelines in preparing your manuscript.
Please note that a copy of this checklist will be published alongside your article.

Abridged guidelines for figures
1. Data
The data shown in figures should satisfy the following conditions:
- the data were obtained and processed according to the field's best practice and are presented to reflect the results of the experiments in an accurate and unbiased manner.
- ideally, figure panels should include only measurements that are directly comparable to each other and obtained with the same assay.
- plots include clearly labeled error bars for independent experiments and sample sizes. Unless justified, error bars should not be shown for technical replicates.
- if n<5, the individual data points from each experiment should be plotted. Any statistical test employed should be justified.
- Source Data should be included to report the data underlying figures according to the guidelines set out in the authorship guidelines on Data Presentation.

2. Captions
Each figure caption should contain the following information, for each panel where they are relevant:
- a specification of the experimental system investigated (eg cell line, species name).
- the assay(s) and method(s) used to carry out the reported observations and measurements.
- an explicit mention of the biological and chemical entity(ies) that are being measured.
- an explicit mention of the biological and chemical entity(ies) that are altered/varied/perturbed in a controlled manner.
- the exact sample size (n) for each experimental group/condition, given as a number, not a range;
- a description of the sample collection allowing the reader to understand whether the samples represent technical or biological replicates (including how many animals, litters, cultures, etc.).
- a statement of how many times the experiment shown was independently replicated in the laboratory.
- definitions of statistical methods and measures:
  - common tests, such as t-test (please specify whether paired vs. unpaired), simple χ^2 tests, Wilcoxon and Mann-Whitney tests, can be unambiguously identified by name only, but more complex techniques should be described in the methods section;
  - are tests one-sided or two-sided?
  - are there adjustments for multiple comparisons?
  - exact statistical test results, e.g., P values = x but not P values < x;
  - definition of 'center values' as median or average;
  - definition of error bars as s.d. or s.e.m.

Please complete ALL of the questions below.
Select "Not Applicable" only when the requested information is not relevant for your study.

Materials

Newly Created Materials	Information included in the manuscript?	In which section is the information available? (Reagents and Tools Table, Materials and Methods, Figures, Data Availability Section)
New materials and reagents need to be available; do any restrictions apply?	Yes	Materials and Methods. No restrictions, newly generated transgenic lines will be shared with other laboratories.

Antibodies	Information included in the manuscript?	In which section is the information available? (Reagents and Tools Table, Materials and Methods, Figures, Data Availability Section)
For **antibodies** provide the following information:<br>- Commercial antibodies: RRID (if possible) or supplier name, catalogue number and or/clone number<br>- Non-commercial: RRID or citation	Yes	Materials and Methods

DNA and RNA sequences	Information included in the manuscript?	In which section is the information available? (Reagents and Tools Table, Materials and Methods, Figures, Data Availability Section)
**Short novel DNA or RNA including primers, probes**: provide the sequences.	Yes	Materials and Methods; supplementary informations

Cell materials	Information included in the manuscript?	In which section is the information available? (Reagents and Tools Table, Materials and Methods, Figures, Data Availability Section)
**Cell lines:** Provide species information, strain. Provide accession number in repository **OR** supplier name, catalog number, clone number, and/**OR** RRID.	Not Applicable	
**Primary cultures:** Provide species, strain, sex of origin, genetic modification status.	Not Applicable	
Report if the cell lines were recently **authenticated** (e.g., by STR profiling) and tested for mycoplasma contamination.	Not Applicable	

Experimental animals	Information included in the manuscript?	In which section is the information available? (Reagents and Tools Table, Materials and Methods, Figures, Data Availability Section)
**Laboratory animals or Model organisms:** Provide species, strain, sex, age, genetic modification status. Provide accession number in repository **OR** supplier name, catalog number, clone number, **OR** RRID.	Yes	Materials and Methods
**Animal observed in or captured from the field:** Provide species, sex, and age where possible.	Not Applicable	
Please detail **housing and husbandry conditions**.	Yes	Materials and Methods

Plants and microbes	Information included in the manuscript?	In which section is the information available? (Reagents and Tools Table, Materials and Methods, Figures, Data Availability Section)
**Plants:** provide species and strain, ecotype and cultivar where relevant, unique accession number if available, and source (including location for collected wild specimens).	Not Applicable	
**Microbes:** provide species and strain, unique accession number if available, and source.	Not Applicable	

Human research participants	Information included in the manuscript?	In which section is the information available? (Reagents and Tools Table, Materials and Methods, Figures, Data Availability Section)
If collected and within the bounds of privacy constraints report on age, sex and gender or ethnicity for all study participants.	Not Applicable	

Core facilities	Information included in the manuscript?	In which section is the information available? (Reagents and Tools Table, Materials and Methods, Figures, Data Availability Section)
If your work benefited from core facilities, was their service mentioned in the acknowledgments section?	Not Applicable	the facilities were mentionned in the Material and Methods section.

Design

Study protocol	Information included in the manuscript?	In which section is the information available? (Reagents and Tools Table, Materials and Methods, Figures, Data Availability Section)
If study protocol has been **pre-registered, provide DOI in the manuscript**. For clinical trials, provide the trial registration number **OR** cite DOI.	Not Applicable	
Report the **clinical trial registration number** (at ClinicalTrials.gov or equivalent), where applicable.	Not Applicable	

Laboratory protocol	Information included in the manuscript?	In which section is the information available? (Reagents and Tools Table, Materials and Methods, Figures, Data Availability Section)
Provide DOI OR other citation details if **external detailed step-by-step protocols** are available.	Not Applicable	

Experimental study design and statistics	Information included in the manuscript?	In which section is the information available? (Reagents and Tools Table, Materials and Methods, Figures, Data Availability Section)
Include a statement about **sample size** estimate even if no statistical methods were used.	Not Applicable	
Were any steps taken to minimize the effects of subjective bias when allocating animals/samples to treatment (e.g. **randomization procedure**)? If yes, have they been described?	Yes	We work with large numbers of embryos randomly distributed. Not detailed in any section.
Include a statement about **blinding** even if no blinding was done.	Not Applicable	
Describe **inclusion/exclusion criteria** if samples or animals were excluded from the analysis. Were the criteria pre-established?<br><br>If sample or data points were omitted from analysis, report if this was due to attrition or intentional exclusion and provide justification.	Not Applicable	
For every figure, are **statistical tests** justified as appropriate? Do the data meet the assumptions of the tests (e.g., normal distribution)? Describe any methods used to assess it. Is there an estimate of variation within each group of data? Is the variance similar between the groups that are being statistically compared?	Yes	see material and methods, as well as figure legends.

Sample definition and in-laboratory replication	Information included in the manuscript?	In which section is the information available? (Reagents and Tools Table, Materials and Methods, Figures, Data Availability Section)
In the figure legends: state number of times the experiment was **replicated** in laboratory.	Yes	figure legends
In the figure legends: define whether data describe **technical or biological replicates**.	Yes	figure legends

Ethics

Ethics	Information included in the manuscript?	In which section is the information available? (Reagents and Tools Table, Materials and Methods, Figures, Data Availability Section)
Studies involving **human participants**: State details of **authority granting ethics approval** (IRB or equivalent committee(s), provide reference number for approval.	Not Applicable	
Studies involving **human participants**: Include a statement confirming that **informed consent** was obtained from all subjects and that the experiments conformed to the principles set out in the WMA Declaration of Helsinki and the Department of Health and Human Services Belmont Report.	Not Applicable	
Studies involving **human participants:** For publication of **patient photos**, include a statement confirming that consent to publish was obtained.	Not Applicable	
Studies involving experimental **animals**: State details of **authority granting ethics approval** (IRB or equivalent committee(s), provide reference number for approval. Include a statement of compliance with ethical regulations.	Yes	in materials and methods
Studies involving **specimen and field samples:** State if relevant **permits** obtained, provide details of authority approving study; if none were required, explain why.	Not Applicable	

Dual Use Research of Concern (DURC)	Information included in the manuscript?	In which section is the information available? (Reagents and Tools Table, Materials and Methods, Figures, Data Availability Section)
Could your study fall under dual use research restrictions? Please check biosecurity documents and list of **select agents and toxins** (CDC): https://www.selectagents.gov/sat/list.htm	Not Applicable	
If you used a select agent, is the security level of the lab appropriate and reported in the manuscript?	Not Applicable	
If a study is subject to dual use research of concern regulations, is the name of the **authority granting approval and reference number** for the regulatory approval provided in the manuscript?	Not Applicable	

Reporting

The MDAR framework recommends adoption of discipline-specific guidelines, established and endorsed through community initiatives. Journals have their own policy about requiring specific guidelines and recommendations to complement MDAR.

Adherence to community standards	Information included in the manuscript?	In which section is the information available? (Reagents and Tools Table, Materials and Methods, Figures, Data Availability Section)
State if relevant guidelines or checklists (e.g., **ICMJE, MIBBI, ARRIVE, PRISMA**) have been followed or provided.	Not Applicable	
For **tumor marker prognostic studies,** we recommend that you follow the **REMARK** reporting guidelines (see link list at top right). See author guidelines, under 'Reporting Guidelines'. Please confirm you have followed these guidelines.	Not Applicable	
For **phase II and III randomized controlled trials**, please refer to the **CONSORT** flow diagram (see link list at top right) and submit the CONSORT checklist (see link list at top right) with your submission. See author guidelines, under 'Reporting Guidelines'. Please confirm you have submitted this list.	Not Applicable	

Data Availability

Data availability	Information included in the manuscript?	In which section is the information available? (Reagents and Tools Table, Materials and Methods, Figures, Data Availability Section)
Have **primary datasets** been deposited according to the journal's guidelines (see 'Data Deposition' section) and the respective accession numbers provided in the Data Availability Section?	Not Applicable	
Were **human clinical and genomic datasets** deposited in a public access-controlled repository in accordance to ethical obligations to the patients and to the applicable consent agreement?	Not Applicable	
Are **computational models** that are central and integral to a study available without restrictions in a machine-readable form? Were the relevant accession numbers or links provided?	Not Applicable	
If publicly available data were reused, provide the respective **data citations in the reference list**.	Not Applicable	
